# White matter predictors of cerebellar tDCS treatment effects in aphasia rehabilitation

**DOI:** 10.3389/fneur.2026.1659337

**Published:** 2026-02-20

**Authors:** Micah A. Johnson, Zafer Keser, Becky Lammers, Myra J. Sydnor, Jamie L. Murter, Patrick Sadil, Yiyang Zhang, John E. Desmond, Argye E. Hillis, Martin A. Lindquist, Rajani Sebastian

**Affiliations:** 1 Department of Biostatistics, Bloomberg School of Public Health, Johns Hopkins University, Baltimore, MD, United States; 2 Department of Physical Medicine and Rehabilitation, School of Medicine, Johns Hopkins University, Baltimore, MD, United States; 3 Department of Neurology, Mayo Clinic, Rochester, MN, United States; 4 Department of Psychology, University of California–Los Angeles, Los Angeles, CA, United States; 5 Department of Neurology, School of Medicine, Johns Hopkins University, Baltimore, MD, United States; 6 Department of Cognitive Science, Krieger School of Arts and Sciences, Johns Hopkins University, Baltimore, MD, United States

**Keywords:** aphasia, cerebellum, diffusion tensor imaging, language, stroke, tDCS, white matter

## Abstract

**Introduction:**

Cerebellar transcranial direct current stimulation (tDCS) combined with language therapy can aid in chronic aphasia recovery, but the neural mechanisms and biomarkers of treatment efficacy remain uncertain.

**Methods:**

In this secondary analysis of data from a previously conducted clinical trial, we used a randomized, double-blind, sham-controlled, within-subject crossover design with a study sample of 19 participants with post-stroke aphasia. We assessed the degree to which baseline properties of cerebro-cerebellar white matter tracts can predict or moderate longitudinal treatment effects at three time points: post-treatment, 2 weeks post-treatment, and 2 months post-treatment. Tract properties were measured by fractional anisotropy (FA) and mean diffusivity (MD) from diffusion tensor imaging (DTI). We also tested whether there are differential effects between trained and untrained language tasks and between cerebellar tDCS polarity (anodal and cathodal).

**Results:**

Baseline measures of tracts connecting the left lesioned cortex to the right posterolateral cerebellum (stimulation target) influenced treatment gains for untrained tasks, relative to sham control. In contrast, for the trained task, treatment gains were influenced by baseline measures of tracts connecting the non-stimulated left cerebellum with the contralateral right cerebral cortex. Although there were no consistent effects from cerebellar tDCS polarity, a highly consistent pattern emerged across all tasks and tracts. Specifically, language improvements were predicted by a baseline tract profile (i.e., higher FA and lower MD) typically associated with higher white matter integrity, especially within the context of stroke-induced white matter decline.

**Discussion:**

These findings corroborate the potential for baseline tract properties as a biomarker of treatment efficacy and support the notion that adjuvant (cerebellar tDCS + language) therapy preferentially benefits individuals with relatively preserved structural connections within functionally relevant networks.

**Clinical trial registration:**

ClinicalTrials.gov, identifier (NCT02901574).

## 1 Introduction

Aphasia is a devastating outcome of stroke. The most widespread current rehabilitation approach for aphasia is speech and language therapy; however, many individuals with post-stroke aphasia still struggle to achieve satisfactory language improvements. Transcranial direct current stimulation (tDCS) shows promise as a complementary treatment to traditional speech and language therapy [see reviews by (1–3)]. Transcranial direct current stimulation (tDCS) is a safe, non-invasive neuromodulation technique that applies low-intensity currents (1–4 mA) between electrodes mounted on the scalp. This current depolarizes or hyperpolarizes neuronal resting membrane potentials and thereby alters cortical excitability (4, 5). Generally, the cortical excitability is increased by anodal tDCS while it is decreased by cathodal tDCS over the same area. Experimental and human studies suggest tDCS elicits long-lasting, polarity-dependent changes in cortical excitability [e.g., (4)]. The after-effects of tDCS are mediated by multiple mechanisms that modify synaptic efficacy similar to those underlying long-term potentiation (LTP) and long-term depression (LDP) of synaptic activity (6–10). Further, synaptic efficacy is most effectively modulated when stimulation coincides with ongoing behavior (e.g., task practice) (11, 12). These state-and activity-dependent principles help explain why pairing tDCS with behavioral therapy can yield additive or synergistic benefits in neurorehabilitation.

The most frequently used approach for tDCS treatment in post-stroke aphasia is stimulation of the intact left hemisphere cortical regions (13–18). An emerging line of work targets the cerebellum [for review, see (19, 100, 101)], particularly the right cerebellum as a nontraditional node for modulating the language network in aphasia. The rationale for stimulating the cerebellum is derived from neuroanatomical, imaging and clinical work including the cerebellar cognitive affective syndrome framework, which establishes the cerebellum’s contributions to higher-order cognition and language functions (20–24). The right cerebellum is anatomically positioned to influence left-hemisphere language circuits via robust, crossed cerebro-cerebellar pathways (25–27). Studies show that cortical projections to pontine nuclei enter the cerebellum via the middle cerebellar peduncle, while Purkinje cell output modulates deep cerebellar nuclei which project through the superior cerebellar peduncle and thalamus back to frontal, parietal, and temporal language areas (28). Cerebellar tDCS is thought to alter Purkinje cell firing and dentate-thalamo-cortical output, thereby shaping the excitability and plasticity of the contralateral cortical regions [for a detailed review see (29)]. Clinically, several studies report that right cerebellar tDCS combined with language therapy improves naming and functional communication in post-stroke aphasia, motivating a closer look at which individuals benefit most (30–34).

Treatment response to neuromodulation varies widely across individuals, and is influenced by diverse factors including demographics (35), lesion characteristics (36–39), baseline aphasia severity (40), and baseline neuroanatomical differences in tissue integrity and organization across relevant gray matter or white matter regions (41–43). White matter architecture may be especially pertinent for cerebellar stimulation, given its reliance on long-range cerebro-cerebellar loops. In our recent work using diffusion tensor imaging (DTI), we found that people with post-stroke aphasia show reduced white matter integrity in tracts connecting the right cerebellum and the lesioned left hemisphere, and that lower integrity in these pathways was associated with poorer picture naming skills (44). Complementary evidence implicates cerebellar peduncle microstructure and right cerebellar gray matter atrophy in aphasia severity and therapy gains (45, 46), paralleling broader findings that neuroanatomical biomarkers predict aphasia severity and responsiveness to treatment. Despite this convergence, no study has directly tested whether baseline properties of cerebro-cerebellar tracts specifically predict or moderate the efficacy of cerebellar tDCS in post-stroke aphasia.

Addressing this gap, the present exploratory study asks whether baseline DTI-derived fractional anisotropy (FA) and mean diffusivity (MD) in cerebro-cerebellar pathways predict or moderate the effects of right cerebellar tDCS, delivered in conjunction with computerized language therapy, on longitudinal language outcomes (post-treatment, 2 weeks post-treatment, and 2 months post-treatment) relative to sham. Our primary aim was to identify tract-specific predictors of treatment effects for the trained outcome (trained naming) and for generalization (untrained naming and functional communication). Based on our prior behavioral and imaging findings, we hypothesized that tracts connecting the stimulated right cerebellum to left-hemisphere language cortex and tracts connecting the left-hemisphere language cortex to the stimulated right cerebellum will be the most informative biomarkers, with greater integrity conferring stronger treatment-related gains. As a secondary aim, we compared anodal and cathodal cerebellar stimulation, testing whether polarity-dependent differences reported previously translate into distinct predictive profiles. We hypothesized that cathodal cerebellar tDCS may be more likely to show treatment and predictive effects, based on previous findings (31, 33).

## 2 Methods

### 2.1 Study design

This study utilized a within-subject crossover design including randomization, double blinding, and sham control. Participants took part in 2 intervention phases of 15 treatment sessions (3–5 sessions per week) with tDCS + computerized aphasia therapy and 15 sessions with sham + computerized aphasia therapy, or the opposite order. Each intervention phase was separated by a washout period of 2 month to minimize potential carry over effects. Eligible participants (see screening criteria in next section) were randomly assigned to the tDCS polarity group (anode or cathode) using block randomization (1:1 ratio). Within each group, participants were also randomized and counterbalanced on the order of interventional phases (either tDCS first then sham, or sham first then tDCS). Each phase involved computerized aphasia treatment and either real tDCS or sham, depending on phase order. All procedures were approved by the Johns Hopkins Medicine Institutional Review Board and participants provided written informed consent. The study was registered with ClinicalTrials.gov (NCT02901574).

### 2.2 Participants

A total of 19 participants from our previous studies (33, 47) who had baseline MRI were included in this study. See Supplementary Figure S1 for a participant flow diagram. Participants were right-handed prior to the stroke, adult English speakers, at least 6 months post-stroke, and diagnosed with aphasia using the Boston Diagnostic Aphasia Examination (48). In this project we recruited only chronic stroke patients (>6 months post-stroke) to avoid the confounding effects of spontaneous recovery, which is most prominent in the acute and subacute phases following stroke. Exclusion criteria included the following: previous neurological or psychiatric diagnosis, right cerebellar lesions, seizures during the previous 12 months, brain surgery, metal in the head, uncorrected visual or hearing loss, scalp sensitivity, greater than 80% naming accuracy on the Philadelphia Naming Test (49), medications that lower the seizure threshold, or *N*-methyl-d-aspartate antagonists (Table 1).

**Table 1:** Demographics of participants. Group refers to tDCS polarity (cathode or anode). Stimulation Order refers to which treatment phase (tDCS or sham) occurred first. BDAE refers to Boston Diagnostic Aphasia Examination.

ID	Group	Age	Sex	Education (years)	Stroke type	Time post-stroke (months)	Aphasia type	Aphasia severity (BDAE)	Phase order	Lesion load
P1	Cathode	79	M	22	Ischemic	8	Fluent	3	tDCS first	1.57
P2	Anode	53	M	18	Ischemic	12	Fluent	3	Sham first	0.04
P3	Cathode	44	M	18	Ischemic	93.5	Fluent	2	Sham first	6.31
P4	Anode	65	M	18	Ischemic	25	Nonfluent	2	tDCS first	3.00
P5	Cathode	64	M	16	Hemorrhage	11.5	Fluent	4	Sham first	1.35
P6	Cathode	69	M	12	Ischemic	168	Fluent	3	tDCS first	13.53
P7	Anode	74	M	16	Ischemic	6.5	Fluent	3	tDCS first	4.50
P8	Anode	47	M	18	Ischemic	64.5	Nonfluent	2	Sham first	6.15
P9	Anode	78	F	16	Ischemic	44	Fluent	2	tDCS first	7.88
P10	Anode	58	M	15	Ischemic	83	Nonfluent	3	tDCS first	2.69
P11	Anode	67	M	10	Ischemic	26.5	Nonfluent	1	Sham first	5.91
P12	Cathode	66	M	14	Ischemic	6	Nonfluent	0	tDCS first	2.63
P13	Cathode	79	M	18	Hemorrhage	37	Fluent	4	Sham first	0.16
P14	Cathode	64	F	12	Ischemic	57	Nonfluent	2	tDCS first	2.68
P15	Cathode	74	M	16	Ischemic	6	Fluent	3	Sham first	2.72
P16	Anode	79	M	18	Hemorrhage	17	Nonfluent	2	Sham first	0.03
P17	Anode	71	M	18	Ischemic	123	Fluent	3	Sham first	7.04
P18	Cathode	59	M	13	Ischemic	118	Nonfluent	3	Sham first	6.99
P19	Cathode	58	M	16	Ischemic	47	Nonfluent	2	tDCS first	9.57

Group refers to tDCS polarity (cathode or anode). Stimulation Order refers to which treatment phase (tDCS or sham) occurred first. BDAE refers to Boston Diagnostic Aphasia Examination.

### 2.3 Language assessment and procedures

For each phase, participants were assessed at four timepoints: before start of treatment (pre-treatment), at end of treatment (post-treatment), 2 weeks after treatment completion (2-weeks post-treatment), and 2 months after treatment completion (2-months post-treatment). The 2-months post-treatment assessment for Phase 1 was also used as the pre-treatment assessment for Phase 2. Aphasia severity was assessed using the Boston Diagnostic Aphasia Examination (48). Outcome variables included a set of 80 trained naming items (Naming 80) and Philadelphia Naming Test [PNT, untrained naming, (49)]. A Speech-Language Pathologist, who was blinded to the participant’s random assignments, both video recorded and scored the naming tasks. Additional outcome variables included functional communication. Functional communication was assessed using the American Speech-Language-Hearing Association Functional Assessment of Communication Skills for Adults (ASHA-FACS) (50). ASHA-FACS contains two rating scales: the Scale of Communication Independence (CI) and the Scale of Qualitative Dimensions of Communication (QDC). The CI scale is a 7-point scale used to rate participant’s level of assistance needed to complete a functional communication task (e.g., making needs or wants known, understanding basic printed materials, etc.). The QDC scale is a 5-point scale used to rate participant’s functional communication as it relates the adequacy, appropriateness, promptness, and communication sharing. Please see Sebastian et al. (33) and Kim et al. (47) for details regarding the naming and functional communication measures.

### 2.4 Cerebellar stimulation

Cerebellar transcranial direct current stimulation (tDCS) was delivered using a constant-current stimulator (Soterix Medical 1 × 1 clinical trials device) at 2 mA for 20 min through two 5 × 5 cm saline-soaked sponge electrodes with carbon rubber pads. The active electrode was centered over the right cerebellar hemisphere, 1 cm inferior and 4 cm lateral to the inion, a scalp coordinate widely used in cerebellar tDCS to preferentially engage the lateral cerebellar cortex involved in cognitive and language processing (32, 51–54). This coordinate is best considered an approximation of the projection of lateral cerebellar regions (including, but not limited to, lobule VII) onto the scalp. The reference electrode was placed on the right shoulder/deltoid. The active and reference electrodes had opposite polarity (either anode/cathode or cathode/anode) depending on the polarity group. To characterize the electric field distribution of this montage, we previously conducted finite element modeling using a high-resolution T1 MRI scan segmented into 11 tissue compartments (32). The modeling results indicated that maximum electric field amplitude (0–1.2 V/m) was generated in the right cerebellum with some spread to the left cerebellum, but without spread to adjacent occipital cortex or other cortical areas, confirming that current flow was primarily concentrated within the right cerebellar tissue.

### 2.5 Randomization and blinding

All participants and study team researchers were blinded to the phase order (tDCS or sham first) via a 6-digit blinded code for initiation of the stimulation sequence. To blind participants during the sham condition, the first 30 s involved a ramp-up (gradual increase) and ramp-down (gradual decrease) of real stimulation, which was insufficient for neuromodulation but sufficient to induce the same scalp sensations as real tDCS. Please see Sebastian et al. (33) for details.

### 2.6 Computerized aphasia treatment

The computerized aphasia treatment was developed by Fridriksson et al. (14, 15, 55) and involved matching pictures depicting common objects with words that were heard and seen (the face of the speaker below the nose is shown on the computer screen). This treatment includes all aspects of lexical-semantic processing and has been demonstrated to improve naming abilities of individuals with post-stroke aphasia with diverse causes of naming deficits (55). Please see Sebastian et al. (33) for details. During the study, participants were freely allowed to also receive standard of care rehabilitation treatment for stroke-related deficits. Active and sham tDCS was administered for the first 20 min of the computerized aphasia treatment.

### 2.7 Neuroimaging

#### 2.7.1 MRI acquisition and preprocessing

Scans were acquired on a Phillips 3 T scanner at the F. M. Kirby Center located at the Kennedy Krieger Institute. High resolution structural brain data was acquired with a 3D sagittal T1-weighted magnetization prepared rapid acquisition with gradient echo (MPRAGE) sequence using the following parameters: slice thickness = 1 mm with no gaps, voxel size = 1 mm^3^, field of view (FOV) = 256 mm × 256 mm × 180 mm, matrix size = 256 mm × 256 mm, TR = 2,000, TE = 3 ms. T2 -weighted structural data at each timepoint were also acquired with the following parameters: slice thickness = 2 mm with no gaps, FOV = 212 mm × 212 mm × 154 mm, matrix size = 256 mm × 256 mm, TR = 4,171, TE = 12 ms. Diffusion-weighted imaging (DWI) data were acquired with the following parameters: in-plane resolution = axial, slice thickness = 2.2 mm with no gap, FOV = 212 mm × 212 mm × 154 mm, matrix size = 256 mm × 256 mm, *b* = 700 s/mm^2^, and 1 non-diffusion weighted (b0) image followed by 32 diffusion-weighted gradients. For most participants, when time permitted, the DWI sequence was acquired twice with identical parameters, and the highest quality version was selected for all subsequent procedures (see Diffusion Preprocessing below). All data were initially prepared by first converting from DICOM to NIFTI format using dcm2niix (56) and then re-organized according to the Brain Imaging Data Structure (BIDS) standard (57).

#### 2.7.2 Lesion load

Lesion masks were manually drawn by a trained neurologist (Z. K.) on the T2 image collected at the first timepoint. Lesion load (LL) was calculated as the proportion of total lesion volume to intracranial volume and was included as a covariate in all analyses.

#### 2.7.3 Diffusion preprocessing and tractography

All diffusion procedures were performed in DSI Studio (58, 59) adapted from Keser et al. (44). Initial quality control was conducted on one or both DWI scans, when applicable, to select the highest quality data based on the number of usable DWI gradients after removing any bad slices/gradients, as well as the degree of neighboring voxelwise correlations, and accuracy of the b-table orientation (which was reoriented if necessary). All DWI data were then resampled to isotropic (1 mm^3^) resolution. Motion correction was conducted by spatially aligning all DWI gradients to the b0 image and eddy-current distortions were corrected by applying FSL’s EDDY (60). The DWI data were reconstructed into diffusion tensor imaging (DTI) data by applying a tensor model. The DTI data were used for tractography as well as tractometry based on two standard metrics: fractional anisotropy (FA) and mean diffusivity (MD). Finally, each participants’ FA image was nonlinearly registered to the ICBM152_adult_FA template so that masks in standard (MNI) space could be used for tractography in native space.

**Figure 1:**
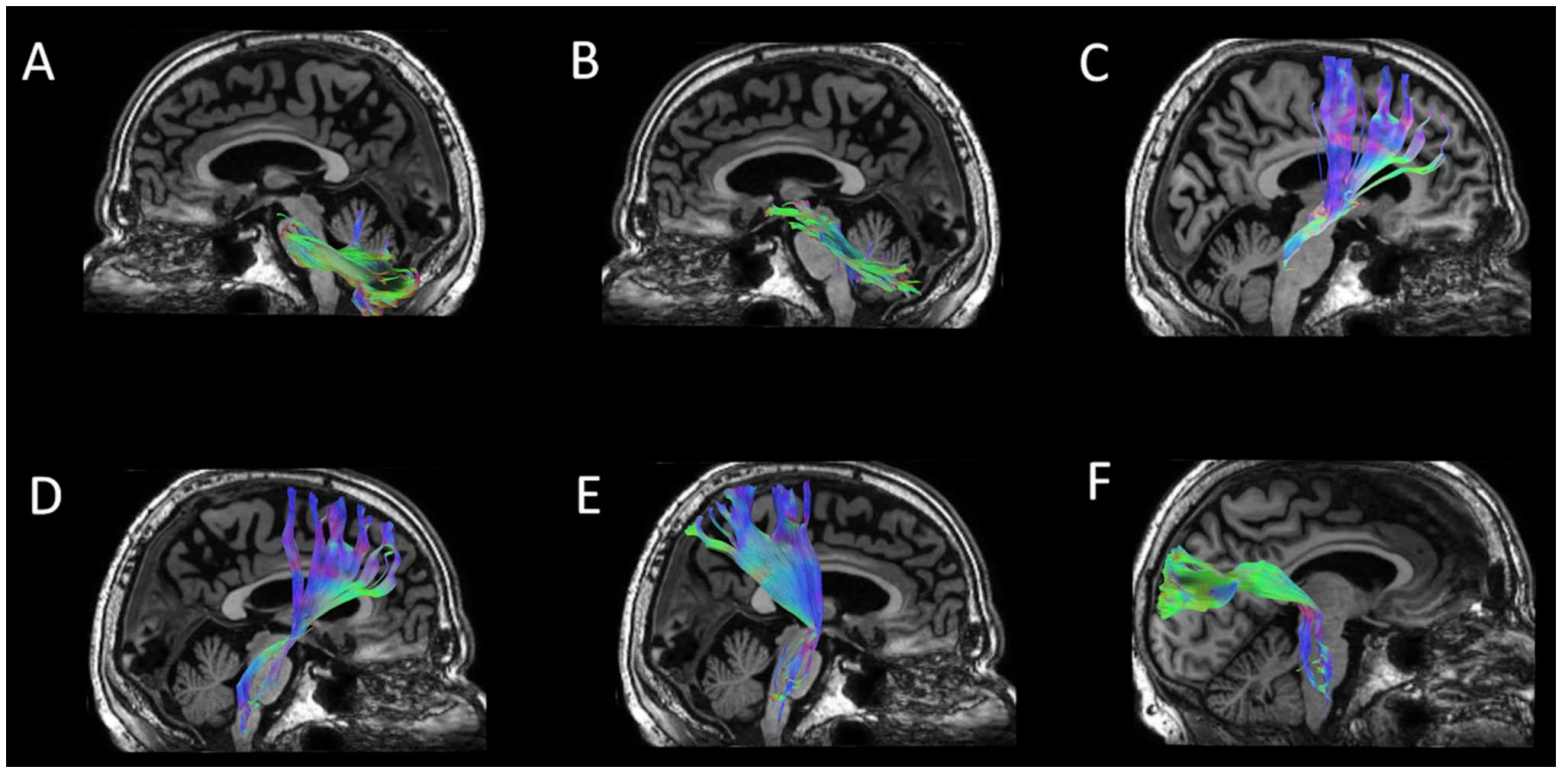
Example 3D illustrations of cerebro-cerebellar tracts estimated with tractography where color coding indicates orientation and type of fibers: blue (mostly projection fibers along the *z* dimension), green (mostly association fibers along the *y* dimension), and red (mostly commisural fibers along the *y* dimension). **(A)** Middle cerebral peduncle (MCP). **(B)** Superior cerebral peduncle (SCP). **(C)** Dentato-rubro-thalamic tract which combines with the contralateral SCP **(B)** to create the dentatorubrothalamocortical tract (DRTC). **(D)** Frontal corticopontine tract (frontal CPT) which combines with the contralateral MCP (A) to create the frontopontocerebellar (FPC) tract. **(E)** Parietal corticopontine tract (parietal CPT) which combines with the contralateral MCP (A) to create the parietopontocerebellar (PPC) tract. **(F)** Occipital corticopontine tract (occipital CPT) which combines with the contralateral MCP (A) to create the occipitopontocerebellar (OPC) tract.

Diffusion tractography in native space was performed using DSIStudio’s AutoTrack procedure, a tract template-based approach that involves a semi-automated brute force method, atlas-defined regions-of-interest (ROIs), and fiber assignment by the continuous tracking (FACT) algorithm (61, 62). In this study we examined the two major pathways connecting the cerebellum and the cortex: (1) descending tracts connecting the left cerebral cortex to the right cerebellum and the right cerebral cortex to the left cerebellum, and (2) ascending tracts connecting the right cerebellum to the left cerebral cortex and the left cerebellum to the right cerebral cortex. In the first step, the following tracts were estimated in each hemisphere: middle cerebral peduncle (MCP), superior cerebral peduncle (SCP), three corticopontine tracts (CPT) including frontopontine (frontal CPT), parietopontine (parietal CPT), and occipitopontine (occipital CPT), and the non-decussating dentatorubrothalamo-cortical tract (nd-DRTC). In the second step, several composite tracts were created. The SCP was combined with the contralateral nd-DRTC to create the full dentatorubrothalamocortical tract (DRTC). The MCP was combined with the contralateral CPT tracts to create the frontopontocerebellar (FPC), parietopontocerebellar (PPC), and occipitopontocerebellar (OPC) tracts. See Figure 1 for example illustrations of these tracts.

These specific tracts were chosen for the following reasons. They are integral parts of the two major pathways between cerebellum and cortex, thereby contributing to the cerebellum’s role in higher-order cognition and language (20–28). Indeed, these tracts and pathways have known relevance to diverse language functions (21, 22, 45, 63–68). We also previously reported on the relevance of these tracts to aphasia and naming at baseline (44), so here we extend the investigation to understanding how baseline properties of these specific tracts may influence cerebellar tDCS treatment effects.

### 2.8 Statistical analysis

#### 2.8.1 Variables and models

In this within-subject design, there were two treatment conditions (tDCS, sham) each with up to four timepoints (pre-treatment, post-treatment, 2-weeks post-treatment, 2-months post-treatment) for the four language outcome variables (Naming 80, PNT, ASHA-FACS CI, and QDC) and baseline measures of white matter tracts. For all analyses predicting language changes, each post-treatment timepoint was normalized relative to the pre-treatment timepoint as either a percentage change [e.g., ((post–pre)/pre)*100] or a difference score (e.g., post–pre) if the pre score was zero, resulting in a final Timepoint factor with three conditions relative to baseline (post-treatment or post-tx, 2-weeks post-treatment or 2-wks post-tx, and 2-months post-treatment or 2-mos post-tx).

The primary research question investigated the degree to which white matter tracts at baseline predicted differential treatment effects on language outcomes. Tract measures at baseline (i.e., the first pre-treatment timepoint, either before tDCS or sham, depending on random assignment of the within-subject order) included average FA and MD values extracted from the primary cerebrocerebellar tracts of interest (bilateral FPC, OPC, PPC, DRTC) as well as the separate tract segments (bilateral cerebral peduncles, MCP and SCP, and bilateral CPTs). Normalized language changes were used as the dependent variable (DV) in separate regression models assessing the degree to which each tract measure moderated or interacted with the stimulation effects by timepoint (Treatment × Timepoint × Tract) or across timepoints (Treatment × Tract). Additional models were also included to assess if tract measures predicted overall language changes from the computerized aphasia therapy by averaging across treatment conditions for each timepoint (Treatment + Timepoint × Tract) or across timepoints as a main effect (Treatment + Timepoint + Tract). For any significant effects from the composite tracts (e.g., left FPC), the results from the constituent tract segments (e.g., left frontal CPT and right MCP) are also reported to help clarify how the effect occurred. Secondary research questions assessed potential treatment effects on language with separate models testing interaction (Treatment × Timepoint) or main effects (Treatment + Timepoint) on the normalized language changes as the DV. For potential effects from tDCS polarity (anode or cathode) on treatment effects or baseline prediction, all above analyses were performed separately within each polarity group in order to at least qualitatively compare differential effects in lieu of quantitative comparison due to insufficient sample size for the higher-order interactions (e.g., Treatment × Timepoint × tDCS group). Finally, potential crossover order effects (tDCS first vs. sham first) influencing the treatment effects on language outcomes were assessed with an additional model (Treatment × Order).

All analyses were conducted with general linear models (GLM) in R Studio (69, 70) using the following packages: *glmmTMB* (71), *emmeans* or *emtrends* (72) for *post hoc* tests, *eff_size* (72) for Cohen’s d effect sizes (73) interpreted by standard convention (around 0.3 for small, 0.5 for medium, or 0.8 for large), and *DHARMa* (74) for model diagnostics. Based on extensive model diagnostics, no random effects were included in any models, due to lack of convergence or poor model fit, and a cube root transformation was applied to each language DV in order to reduce or eliminate any violations of standard assumptions (e.g., linearity, independence of errors, homoscedasticity, dispersion, outliers, or normality). Three covariates were included in all analyses when appropriate to control for their potential effects: lesion load, aphasia severity (BDAE), and the within-subject order of stimulation phases (e.g., active tDCS or sham first).

#### 2.8.2 Statistical significance and multiple comparisons correction

Given the exploratory nature of this study combined with the large number of analyses involving different language tasks, bilateral tracts, diffusivity measures, and interaction/main effects, we applied a global *p-*value threshold (*p* < 0.01) across all models for determining statistical significance, and we report *p* values between 0.01 and 0.05 as marginally significant and/or trend or trend-level effects. If any interaction did not pass statistical significance, we still reported *post hoc* comparisons between nested levels (e.g., treatment effects by timepoint) for exploratory purposes given *a priori* interest in all potential effects. We adjusted for multiple comparisons among the post hoc comparisons separately for each interaction model using a false discovery rate (FDR) correction (*q* = 0.05). When reporting simple main effects, such as mean changes or slopes for separate treatment levels (e.g., active tDCS) or treatment by timepoint levels (e.g., active tDCS at 2-weeks post-treatment), we determined reliability of these effects based on whether the confidence interval of the mean change or slope included zero, and when the confidence interval was close but not passing this criterion, we refer to these as trend-level effects.

## 3 Results

We briefly present the behavioral effects of tDCS versus sham on trained naming (Naming 80), untrained naming (PNT), and functional communication skills (ASHA FACS CI and QDC scales) at post-treatment, 2 weeks post-treatment, and 2 months post-treatment. Subsequently, we present results that address the question of which white matter tracts predicted (i.e., moderated) the treatment effects. Results for the tDCS polarity groups combined (anode and cathode) are shown first followed by group specific results (anode or cathode). The behavioral results have been previously reported in detail in Sebastian et al. (33) and Kim et al. (47).

### 3.1 Behavioral treatment effects on language outcomes

#### 3.1.1 Summary

Computerized aphasia therapy combined with cerebellar tDCS stimulation resulted in treatment effects of larger improvements, relative to sham control, for two language outcomes: PNT Untrained (for tDCS polarity groups combined, with similar trends for anodal and cathodal groups) and ASHA-FACS QDC (for tDCS polarity groups combined, and also separately for the anodal group and especially the cathodal group). The treatment gains began immediately (post-tx) and were maintained over time (2-wks and 2-mo post-tx), as indicated by the lack of significant interactions between treatment and timepoints. The other language outcomes (Naming 80 and ASHA-FACS CI scale) showed no evidence of treatment effects with polarity groups combined or separate. Table 2 presents the Treatment × Timepoint interaction and Treatment main effects for the combined tDCS polarity groups, along with *post hoc* test results (see Supplementary Appendix 2A for complete details). Results for each polarity group analyzed separately are provided in Supplementary Appendix 2B. To assess potential carryover or phase-order effects, we conducted additional Treatment × Order models for each language outcome using the combined groups. None of these interactions were statistically significant (see Supplementary Appendix 2C). The behavioral results have been previously reported in Sebastian et al. (33) and Kim et al. (47).

**Table 2:** Results of the GLM testing for interaction between Treatment (Tx: tDCS vs. sham) and Timepoint (Tpt: post-treatment, 2-weeks post-tx, 2-months post-tx), or main effect of Treatment, conducted separately for each language measure as the dependent variable (DV): Naming 80 (trained naming), Philadelphia Naming Test (PNT, untrained naming), American Speech-Language-Hearing Association Functional Assessment of Communication Skills for Adults, Scale of Communication Independence (ASHA-FACS CI), American Speech-Language-Hearing Association Functional Assessment of Communication Skills for Adults, Scale of Qualitative Dimensions of Communication (ASHA-FACS QDC). Δ*M* refers to the %mean change, *SE* refers to the % standardized error, 95% CI refers to the lower and upper bounds of the 95% confidence interval, *t* refers to the t-test value with corresponding *p* value, and *d* refers to Cohen’s d estimate of effect size (with corresponding lower and upper bounds of the 95% confidence interval).

								tDCS			Sham			tDCS vs. Sham			
DV	Term	df1	df2	*F*	*p*	*R* ^2^	Tpt	ΔM	SE	95% CI	ΔM	SE	95% CI	*t*	*p*	*d*	95% CI
Naming 80	Tx	1	99	0.28	0.5985	0.20	All	50.80	7.58	[35.80, 65.90]	56.60	7.83	[41.00, 72.10]	−0.53	0.5985	−0.10	[−0.49, 0.28]
	Tx × Tpt	2	97	0.02	0.9825	0.20	Post-tx	57.00	12.90	[31.40, 82.60]	64.70	13.20	[38.50, 91.0]	−0.42	0.6768	−0.14	[−0.79, 0.52]
							2-wks post-tx	53.20	13.20	[27.00, 79.50]	56.10	13.60	[29.10, 83.20]	−0.15	0.8784	−0.05	[−0.72, 0.62]
							2-mos post-tx	42.30	13.20	[16.10, 68.60]	48.80	13.60	[21.70, 75.90]	−0.34	0.7342	−0.12	[−0.79, 0.56]
PNT	Tx	1	98	5.37	0.0225	0.06	All	28.74	6.49	[15.85, 41.60]	7.08	6.77	[−6.35, 20.50]	2.32	0.0225	0.45	[0.06, 0.84]
	Tx × Tpt	2	96	0.79	0.4530	0.07	Post-tx	26.21	11.00	[4.45, 48.0]	14.46	11.30	[−7.88, 36.90]	0.75	0.4564	0.25	[−0.41, 0.90]
							2-wks post-tx	20.09	11.30	[−2.26, –42.40]	4.71	11.90	[−19.01, 28.40]	0.94	0.3506	0.32	[−0.36, 1.01]
							2-mos post-tx	40.18	11.30	[17.84, 62.50]	1.97	11.60	[−21.05, 25.0]	2.37	0.0199	0.80	[0.12, 1.48]
ASHA-FACS (CI)	Tx	1	96	0.19	0.6651	0.05	All	26.60	3.76	[19.20, 34.10]	24.30	3.76	[16.90, 31.80]	0.43	0.6651	0.09	[−0.31, 0.48]
	Tx × Tpt	2	94	1.16	0.3168	0.07	Post-tx	28.70	6.13	[16.56, 40.90]	16.10	6.29	[3.56, 28.60]	1.44	0.1526	0.48	[−0.18, 1.13]
							2-wks post-tx	28.00	6.71	[14.66, 41.30]	28.70	6.48	[15.83, 41.60]	−0.08	0.9378	−0.03	[−0.72, 0.67]
							2-mos post-tx	22.60	6.47	[9.71, 35.40]	28.60	6.48	[15.70, 41.50]	−0.66	0.5123	−0.23	[−0.91, 0.46]
ASHA-FACS (QDC)	Tx	1	96	15.18	0.0002	0.24	All	31.70	3.67	[24.36, 38.90]	11.50	3.67	[4.23, 18.80]	3.89	0.0002	0.77	[0.36, 1.17]
	Tx × Tpt	2	94	0.18	0.8354	0.24	Post-tx	31.81	6.04	[19.82, 43.80]	7.93	6.20	[−4.38, 20.20]	2.76	0.0070	0.91	[0.24, 1.58]
							2-wks post-tx	32.46	6.61	[19.33, 45.60]	12.67	6.39	[−0.02, 25.30]	2.16	0.0332	0.75	[0.05, 1.45]
							2-mos post-tx	30.47	6.37	[17.82, 43.10]	14.07	6.39	[1.39, 26.80]	1.82	0.0721	0.62	[−0.06, 1.31]

### 3.2 Baseline cerebrocerebellar tracts predicting language outcomes

#### 3.2.1 Overall summary

Baseline measures (FA, MD) of cerebro-cerebellar tracts predicted or moderated the treatment effects of language gains after tDCS relative to sham. ASHA-FACS scores were predicted mostly by tracts connecting the left cerebral cortex and the stimulated right cerebellum, and the trained task (Naming 80) was predicted mostly by tracts connecting the non-stimulated left cerebellum and the right cerebral cortex. The untrained naming task (PNT) scores showed some evidence of prediction from the non-stimulated left cerebellum (middle cerebellar peduncle) and other evidence of prediction from cerebrocerebellar tracts although specific to only the sham condition. In most cases, higher language improvements after tDCS were predicted by baseline properties typically associated with higher white matter integrity (i.e., higher FA and lower MD). This pattern was consistent for most cerebro-cerebellar tracts predicting ASHA-FACS (CI and QDC) and Naming 80 (trained), but PNT (untrained) showed a more mixed pattern. For the cerebral peduncles, the same pattern was consistent for all tasks except for the ASHA-FACS (QDC) which showed some opposite effects between polarity groups. Results were also similar between cerebral and cerebellar tract segments and also between tDCS polarity (anode vs. cathode) groups (see Supplementary Appendices 3A–5B).

**Figure 2:**
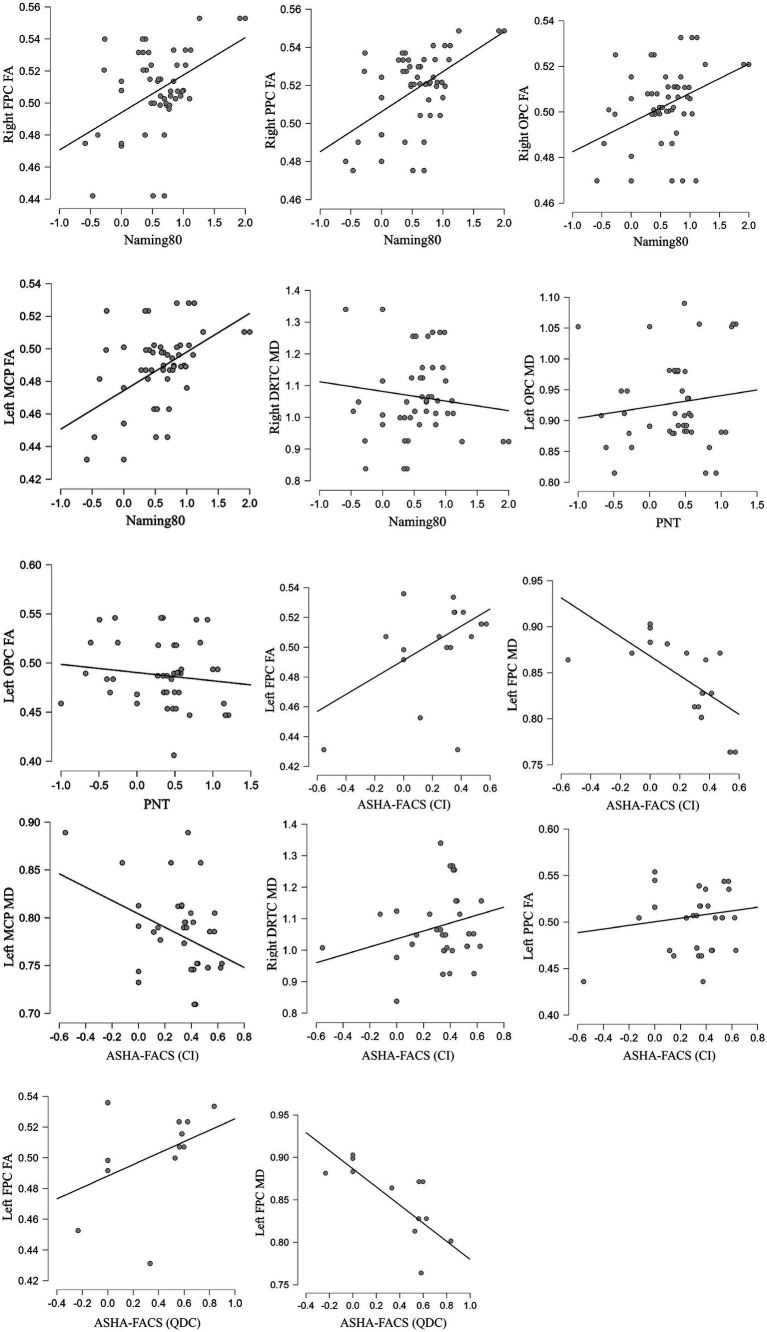
Scatterplots of the main findings for baseline tract predictions of the tDCS effects showing relationships between baseline tract measures and outcome measures changes (averaged across all post-treatment timepoints, cube-root transformations of the percent change relative to pre-treatment baseline). FPC, frontopontocerebellar tract; PPC, parietopontocerebellar tract; OPC, occipitopontocerebellar tract; DRTC, dentatorubrothalamocortical tract; MCP, middle cerebellar peduncle; FA, fractional anisotropy; MD, mean diffusivity; Outcome measures: Naming 80, Philadelphia Naming Test (PNT), American Speech-Language-Hearing Association Functional Assessment of Communication Skills for Adults, Scale of Communication Independence (ASHA-FACS CI), American Speech-Language-Hearing Association Functional Assessment of Communication Skills for Adults, Scale of Qualitative Dimensions of Communication (ASHA-FACS QDC).

See Table 3 for a summary of the baseline prediction results, with tDCS polarity groups combined, for composite tracts or cerebellar peduncles that passed global correction (*p* < 0.01) with reliable linear relationships interpreted by the confidence interval of the beta coefficient not including zero. See Figure 2 for corresponding scatterplot visualizations. For full results of all baseline predictions (Supplementary Appendices 3A–5B).

**Table 3:** Summary of the baseline prediction results that passed global correction (*p* < 0.01) for each language measure as the dependent variable (DV): Naming 80 (trained naming), Philadelphia Naming Test (PNT, untrained naming), American Speech-Language-Hearing Association Functional Assessment of Communication Skills for Adults, Scale of Communication Independence (ASHA-FACS CI), American Speech-Language-Hearing Association Functional Assessment of Communication Skills for Adults, Scale of Qualitative Dimensions of Communication (ASHA-FACS QDC).

DV	Tract	DTI	Interaction (Treatment * Tract)					tDCS			Sham		
			df1	df2	*F*	*p*	*R* ^2^	*b*	SE	95% CI	*b*	SE	95% CI
Naming 80	Right FPC	FA	1	97	7.44	0.0076	0.26	7.50	2.89	[1.77, 13.23]	−3.50	2.96	[−9.37, 2.37]
	Right PPC	FA	1	97	9.03	0.0034	0.28	12.50	3.66	[5.25, 19.76]	−3.18	3.89	[−10.89, 4.53]
	Right OPC	FA	1	97	8.53	0.0043	0.26	11.93	4.53	[2.94, 20.92]	−6.89	4.92	[−16.65, 2.88]
	Right DRTC	MD	1	90	7.94	0.0059	0.25	−0.69	0.64	[−1.96, 0.58]	1.87	0.72	[0.43, 3.31]
	Left MCP	FA	1	97	7.20	0.0086	0.26	8.85	3.10	[2.69, 15.01]	−3.11	3.41	[−9.88, 3.66]
PNT	Left OPC	FA	1	78	7.74	0.0068	0.19	−2.07	2.15	[−6.34, 2.21]	6.59	2.28	[2.05, 11.13]
		MD	1	78	7.34	0.0083	0.19	0.49	1.11	[−1.73, 2.70]	−3.32	1.13	[−5.57, −1.08]
ASHA-FACS (CI)	Left FPC	FA	1	48	10.18	0.0025	0.32	7.25	1.58	[4.06, 10.43]	1.14	1.53	[−1.94, 4.22]
		MD	1	48	8.48	0.0054	0.33	−5.29	1.15	[−7.59, −2.99]	−1.29	1.13	[−3.57, 0.99]
	Left PPC	FA	1	75	8.03	0.0059	0.13	1.70	1.25	[−0.79, 4.18]	−2.89	1.23	[−5.35, −0.44]
	Right DRTC	MD	1	87	9.18	0.0032	0.12	0.59	0.31	[−0.03, 1.20]	−0.74	0.35	[−1.43, −0.05]
	Left MCP	MD	1	94	14.72	0.0002	0.19	−3.59	0.89	[−5.36, −1.81]	0.81	0.89	[−0.96, 2.57]
ASHA-FACS (QDC)	Left FPC	FA	1	48	12.09	0.0011	0.49	7.17	1.46	[4.24, 10.10]	1.04	1.41	[−1.79, 3.88]
		MD	1	48	11.46	0.0014	0.56	−6.01	0.98	[−7.98, −4.05]	−2.04	0.97	[−3.99, −0.09]

Reliable linear relationships interpreted by the 95% confidence interval (CI) of the beta coefficient (b) not including zero.

#### 3.2.2 Naming 80 (trained naming)

##### 3.2.2.1 tDCS polarity groups combined

Longitudinal gains in the Naming 80 task after active tDCS, relative to sham, were significantly predicted by higher FA at baseline in several composite tracts connecting the right cortex to the left (non-stimulated) cerebellum, specifically for the right FPC [Treatment × Tract: *F*(1, 97) = 7.44, *p* = 0.0076, *R*^2^ = 0.26; active tDCS: *b* = 7.50, SE = 2.89, 95% CI = (1.77, 13.23); sham: *b* = −3.50, SE = 2.96, 95% CI = (−9.37, 2.37)], right PPC [Treatment × Tract: *F*(1, 97) = 9.03, *p* = 0.0034, *R*^2^ = 0.28; active tDCS: *b* = 12.50, SE = 3.66, 95% CI = (5.25, 19.76); sham: *b* = −3.18, SE = 3.89, 95% CI = (−10.89, 4.53)], and right OPC [Treatment × Tract: *F*(1, 97) = 8.53, *p* = 0.0043, *R*^2^ = 0.26; active tDCS: *b* = 11.93, SE = 4.53, 95% CI = (2.94, 20.92); sham: *b* = −6.89, SE = 4.92, 95% CI = (−16.65, 2.88)]. The effects in the composite tracts were driven primarily by the left MCP FA [Treatment × Tract: *F*(1, 97) = 7.20, *p* = 0.0086, *R*^2^ = 0.26; active tDCS: *b* = 8.85, SE = 3.10, 95% CI = (2.96, 15.01); sham: *b* = −3.11, SE = 3.41, 95% CI = (−9.88, 3.66)]. There was also a significant Treatment × Tract interaction involving right DRTC [Treatment × Tract: *F*(1, 90) = 7.94, *p* = 0.0059, *R*^2^ = 0.26] but it resulted from higher MD predicting higher gains for sham [*b* = 1.87, SE = 0.70, 95% CI = (0.43, 3.31)] but without any reliable effect for tDCS [*b* = −0.69, SE = 0.60, 95% CI = (−1.96, 0.58)].

##### 3.2.2.2 Separate tDCS polarity groups

The above effects mostly dissipated when separating anodal and cathodal groups. In the anodal group, none of the tracts reliably predicted active tDCS gains, despite significant Treatment × Tract interactions, which resulted only from sham effects (see Supplementary Appendix 3B). In the cathodal group, longitudinal gains after active tDCS, relative to sham, were significantly predicted by lower MD in the left PPC [Treatment × Tract: *F*(1, 32) = 10.41, *p* = 0.0029, *R*^2^ = 0.37; active tDCS: *b* = −13.60, SE = 4.06, 95% CI = (−21.90, −5.32); sham: *b* = −6.40, SE = 4.46, 95% CI = (−15.50, 2.68)] and by lower MD in the left FPC [Treatment × Tract: *F*(1, 16) = 8.28, *p* = 0.0110, *R*^2^ = 0.50; active tDCS: *b* = −16.00, SE = 4.20, 95% CI = (−24.89, −7.08); sham: *b* = 1.10, SE = 4.20, 95% CI = (−7.81, 10.00)].

#### 3.2.3 PNT (untrained naming)

##### 3.2.3.1 tDCS polarity groups combined

None of the composite tracts or corticopontine tracts significantly predicted PNT gains for active tDCS but some significantly predicted gains for sham (see Supplementary Appendix 3A). Specifically, there was a significant Treatment × Tract interaction involving left OPC FA [*F*(1, 78) = 7.74, *p* = 0.0068, *R*^2^ = 0.19] but it resulted from higher FA predicting higher gains for sham [*b* = 6.59, SE = 2.28, 95% CI = (2.05, 11.13)] but without any reliable effect for tDCS [*b* = −2.07, SE = 2.15, 95% CI = (−6.34, 2.21)]. There was also a significant Treatment × Tract interaction involving left OPC MD [Treatment × Tract: *F*(1, 78) = 7.34, *p* = 0.0083, *R*^2^ = 0.19] but it resulted from lower MD predicting higher gains for sham [*b* = −3.32, SE = 1.13, 95% CI = (−5.57, −1.08)] without any reliable effect for tDCS [*b* = 0.49, SE = 1.11, 95% CI = (−1.73, 2.70)]. Finally, there was a marginally significant interaction effect from right MCP such that lower MD at baseline predicted higher gains for active but not sham [Treatment × Tract: *F*(1, 96) = 4.52, *p* = 0.0360, *R*^2^ = 0.13; active tDCS: *b* = −4.57, SE = 1.57, 95% CI = (−7.68, −1.46); sham: *b* = 0.01, SE = 1.61, 95% CI = (−3.18, 3.20)].

##### 3.2.3.2 Separate tDCS polarity groups

In the anodal group, there was a significant Treatment × Tract interaction involving left PPC MD such that higher MD at baseline predicted higher gains for active but not sham [Treatment × Tract: *F*(1, 38) = 10.23, *p* = 0.0028, *R*^2^ = 0.34; active tDCS: *b* = 6.77, SE = 3.13, 95% CI = (0.44, 13.10); sham: *b* = −2.85, SE = 3.13, 95% CI = (−9.18, 3.49)]. There was also a marginally significant Treatment × Tract interaction involving left PPC FA such that lower FA at baseline trend-level predicted higher gains for active but not sham [Treatment × Tract: *F*(1, 38) = 5.78, *p* = 0.0212, *R*^2^ = 0.28; active tDCS: *b* = −7.09, SE = 3.85, 95% CI = (−14.89, 0.71); sham: *b* = 5.06, SE = 3.85, 95% CI = (−2.74, 12.86)]. The left PPC effects appeared to be driven by a marginally significant interaction from the right MCP segment such that lower MD reliably predicted higher gains for active but not sham [Treatment × Tract: *F*(1, 44) = 6.49, *p* = 0.0144, *R*^2^ = 0.25; active tDCS: *b* = −4.89, SE = 2.25, 95% CI = (−9.44, −0.35); sham: *b* = 2.22, SE = 2.25, 95% CI = (−2.33, 6.76)]. It was also driven by marginally significant interactions involving the left parietal CPT segment such that higher gains for active, but not sham, were predicted by higher MD [Treatment × Tract: *F*(1, 38) = 15.13, *p* = 0.0004, *R*^2^ = 0.39; active tDCS: *b* = 2.78, SE = 0.97, 95% CI = (0.82, 4.75); sham: *b* = −1.77, SE = 0.97, 95% CI = (−3.73, 0.20)] and lower FA [Treatment × Tract: *F*(1, 38) = 5.95, *p* = 0.0195, *R*^2^ = 0.28; active tDCS: *b* = −4.75, SE = 2.44, 95% CI = (−9.68, 0.19); sham: *b* = 3.01, SE = 2.44, 95% CI = (−6.74, 1.63)]. There was also a significant Treatment × Tract interaction involving the right DRTC MD but it was driven by a trend-level prediction of only the sham changes [Treatment × Tract: *F*(1, 38) = 7.63, *p* = 0.0088, *R*^2^ = 0.32; active tDCS: *b* = −2.89, SE = 5.10, 95% CI = (−13.20, 7.43); sham: *b* = −9.53, SE = 5.10, 95% CI = (−19.90, 0.79)].

In the cathodal group, there was a significant Treatment × Tract interaction involving left FPC such that lower MD at baseline predicted higher gains for active but the opposite effect for sham [Treatment × Tract: *F*(1, 15) = 49.62, *p* < 0.0001, *R*^2^ = 0.73; active tDCS: *b* = −9.21, SE = 1.91, 95% CI = (−13.28, −5.13); sham: *b* = 10.01, SE = 1.95, 95% CI = (5.86, 14.16)]. There was a marginally significant Treatment × Tract interaction involving left PPC such that lower MD at baseline predicted higher gains for active but not sham [Treatment × Tract: *F*(1, 31) = 5.53, *p* = 0.0252, *R*^2^ = 0.24; active tDCS: *b* = −7.76, SE = 2.86, 95% CI = (−13.65, −1.86); sham: *b* = 2.18, SE = 3.28, 95% CI = (−4.52, 8.88)]. The left FPC and PPC effects appeared to be driven by lower MD in the left frontal CPT [Treatment × Tract: *F*(1, 15) = 21.10, *p* = 0.0004, *R*^2^ = 0.57; active tDCS: *b* = −2.81, SE = 1.10, 95% CI = (−5.16, −0.45); sham: *b* = 4.20, SE = 1.11, 95% CI = (1.83, 6.57)] and lower MD in left parietal CPT [Treatment × Tract: *F*(1, 31) = 5.35, *p* = 0.0275, *R*^2^ = 0.22; active tDCS: *b* = −4.06, SE = 1.75, 95% CI = (−7.64, −0.49); sham: *b* = 2.00, SE = 1.94, 95% CI = (−1.96, 5.96)].

#### 3.2.4 ASHA-FACS CI scale

##### 3.2.4.1 tDCS polarity groups combined

Longitudinal gains on the ASHA-FACS CI scale after active tDCS, relative to sham, were significantly predicted by the left FPC connecting left cortex to the right stimulated cerebellum such that higher gains were predicted by higher FA [Treatment × Tract: *F*(1, 48) = 10.18, *p* = 0.0025, *R*^2^ = 0.32; active tDCS: *b* = 7.25, SE = 1.58, 95% CI = (4.06, 10.43); sham: *b* = 1.14, SE = 1.53, 95% CI = (−1.94, 4.22)] and lower MD [Treatment × Tract: *F*(1, 48) = 8.48, *p* = 0.0054, *R*^2^ = 0.33; active tDCS: *b* = −5.29, SE = 1.15, 95% CI = (−7.59, −2.99); sham: *b* = −1.29, SE = 1.13, 95% CI = (−3.57, 0.99)]. There was a significant Treatment × Tract interaction involving right DRTC such that higher gains were trend-level predicted by higher MD for active tDCS but reliably predicted by lower MD for sham [Treatment × Tract: *F*(1, 87) = 9.18, *p* = 0.0032, *R*^2^ = 0.12; active tDCS: *b* = 0.59, SE = 0.31, 95% CI = (−0.03, 1.20); sham: *b* = −0.74, SE = 0.35, 95% CI = (−1.43, −0.05)]. There was a significant Treatment × Tract interaction involving left MCP such that higher gains were reliably predicted by lower MD for active tDCS but not sham [Treatment × Tract: *F*(1, 94) = 14.72, *p* = 0.0002, *R*^2^ = 0.19; active tDCS: *b* = −3.59, SE = 0.89, 95% CI = (−5.36, −1.81); sham: *b* = 0.81, SE = 0.89, 95% CI = (−0.96, 2.57)]. There were also marginally significant interactions for left frontal CPT such that higher FA predicted higher gains for active but not sham [Treatment × Tract: *F*(1, 48) = 5.48, *p* = 0.0235, *R*^2^ = 0.26; active tDCS: *b* = 4.16, SE = 1.08, 95% CI = (1.98, 6.33); sham: *b* = 1.31, SE = 1.05, 95% CI = (−0.79, 3.42)].

##### 3.2.4.2 Separate tDCS polarity groups

In the anodal group, none of the tracts reliably predicted active tDCS gains, despite some significant Treatment × Tract interactions, which resulted only from sham effects (see Supplementary Appendix 3B). In the cathodal group, higher gains were significantly predicted by several tract profiles. Higher baseline MD in the right DRTC predicted improvements from active tDCS but the opposite effect for sham [Treatment × Tract: *F*(1, 43) = 13.28, *p* = 0.0007, *R*^2^ = 0.22; active tDCS: *b* = 0.72, SE = 0.31, 95% CI = (0.09, 1.35); sham: *b* = −0.91, SE = 0.34, 95% CI = (−1.59, −0.23)]. Lower baseline MD in the right OPC predicted improvements from active but not sham [Treatment × Tract: *F*(1, 44) = 4.38, *p* = 0.0423, *R*^2^ = 0.11; active tDCS: *b* = −2.96, SE = 1.36, 95% CI = (−5.70, −0.23); sham: *b* = 1.00, SE = 1.36, 95% CI = (−1.73, 3.74)]. Higher FA in the left FPC predicted improvements from active but not sham [Treatment × Tract: *F*(1, 16) = 7.71, *p* = 0.0135, *R*^2^ = 0.49; active tDCS: *b* = 8.71, SE = 2.76, 95% CI = (2.85, 14.56); sham: *b* = 2.83, SE = 2.76, 95% CI = (−3.02, 8.68)]. Improvements after tDCS but not sham were also predicted by lower MD in the left SCP [Treatment × Tract: *F*(1, 44) = 6.81, *p* = 0.0123, *R*^2^ = 0.14; active tDCS: *b* = −0.69, SE = 0.31, 95% CI = (−1.31, −0.07); sham: *b* = 0.55, SE = 0.37, 95% CI = (−0.19, 1.29)] and higher MD in the right SCP [Treatment × Tract: *F*(1, 44) = 7.68, *p* = 0.0081, *R*^2^ = 0.15; active tDCS: *b* = 0.71, SE = 0.33, 95% CI = (0.05, 1.37); sham: *b* = −0.56, SE = 0.35, 95% CI = (−1.26, 0.14)]. Higher FA in the left frontal CPT also predicted active but not sham [Treatment × Tract: *F*(1, 16) = 15.14, *p* = 0.0013, *R*^2^ = 0.61; active tDCS: *b* = 7.63, SE = 1.95, 95% CI = (3.49, 11.78); sham: *b* = 2.96, SE = 1.95, 95% CI = (−1.18, 7.11)].

#### 3.2.5 ASHA-FACS QDC scale

##### 3.2.5.1 tDCS polarity groups combined

Longitudinal gains on the ASHA-FACS QDC scale after active tDCS, relative to sham, were significantly predicted by the left FPC connecting left cortex to the right (stimulated) cerebellum such that higher gains were predicted by higher FA [Treatment × Tract: *F*(1, 48) = 12.09, *p* = 0.0011, *R*^2^ = 0.49; active tDCS: *b* = 7.17, SE = 1.46, 95% CI = (4.24, 10.10); sham: *b* = 1.04, SE = 1.41, 95% CI = (−1.79, 3.88)] and lower MD [Treatment × Tract: *F*(1, 48) = 11.46, *p* = 0.0014, *R*^2^ = 0.56; active tDCS: *b* = −6.01, SE = 0.98, 95% CI = (−7.98, −4.05); sham: *b* = −2.04, SE = 0.97, 95% CI = (−3.99, −0.09)]. These left FPC effects were driven by the left frontal CPT having higher FA [Treatment × Tract: *F*(1, 48) = 22.02, *p* < 0.0001, *R*^2^ = 0.55; active tDCS: *b* = 5.12, SE = 0.90, 95% CI = (3.31, 6.93); sham: *b* = 0.38, SE = 0.87, 95% CI = (−1.37, 2.13)] and lower MD [Treatment × Tract: *F*(1, 48) = 36.62, *p* < 0.0001, *R*^2^ = 0.58; active tDCS: *b* = −3.16, SE = 0.55, 95% CI = (−4.27, −2.05); sham: *b* = 1.05, SE = 0.53, 95% CI = (−0.01, 2.12)]. There was also significant Treatment × Tract interaction involving the right frontal CPT such that higher gains were predicted by higher FA for active but not sham [Treatment × Tract: *F*(1, 94) = 7.66, *p* = 0.0068, *R*^2^ = 0.30; active tDCS: *b* = 2.95, SE = 1.02, 95% CI = (0.93, 4.96); sham: *b* = −0.88, SE = 0.96, 95% CI = (−2.79, 1.03)].

##### 3.2.5.2 Separate tDCS polarity groups

In the anodal group, higher gains were significantly predicted by several tract profiles. Higher baseline FA in the left FPC predicted gains after tDCS but not sham [Treatment × Tract: *F*(1, 24) = 15.79, *p* = 0.0006, *R*^2^ = 0.62; active tDCS: *b* = 11.62, SE = 2.40, 95% CI = (6.66, 16.58); sham: *b* = −0.49, SE = 2.09, 95% CI = (−4.82, 3.82)]. Higher baseline MD in the left MCP predicted gains from active but not sham [Treatment × Tract: *F*(1, 42) = 8.05, *p* = 0.0070, *R*^2^ = 0.41; active tDCS: *b* = 5.28, SE = 1.83, 95% CI = (1.58, 8.98); sham: *b* = −1.19, SE = 1.81, 95% CI = (−4.84, 2.45)]. Higher gains from active tDCS but not sham were also predicted by baseline FA in the right SCP [Treatment × Tract: *F*(1, 42) = 6.95, *p* = 0.0117, *R*^2^ = 0.42; active tDCS: *b* = −5.41, SE = 1.61, 95% CI = (−8.66, −2.16); sham: *b* = −0.11, SE = 1.53, 95% CI = (−3.21, 2.98)] and higher baseline MD in the right SCP [Treatment × Tract: *F*(1, 42) = 13.23, *p* = 0.0007, *R*^2^ = 0.49; active tDCS: *b* = 2.14, SE = 0.50, 95% CI = (1.13, 3.15); sham: *b* = −0.13, SE = 0.46, 95% CI = (−1.07, 0.80)]. Finally, active gains were predicted by frontal CPT having higher FA [Treatment × Tract: *F*(1, 24) = 21.45, *p* = 0.0001, *R*^2^ = 0.64; active tDCS: *b* = 6.15, SE = 1.27, 95% CI = (3.53, 8.78); sham: *b* = −1.36, SE = 1.16, 95% CI = (−3.76, 1.03)] and lower MD [Treatment × Tract: *F*(1, 24) = 26.02, *p* < 0.0001, *R*^2^ = 0.63; active tDCS: *b* = −2.72, SE = 0.77, 95% CI = (−4.32, −1.12); sham: *b* = 2.35, SE = 0.69, 95% CI = (0.93, 3.77)].

In the cathodal group, higher gains were significantly predicted by lower baseline MD in the left FPC for active but not sham [Treatment × Tract: *F*(1, 16) = 167.93, *p* < 0.0001, *R*^2^ = 0.95; active tDCS: *b* = −9.20, SE = 0.51, 95% CI = (−10.27, −8.13); sham: *b* = 0.05, SE = 0.51, 95% CI = (−1.02, 1.12)] and by lower baseline MD in the left PPC for active but not sham [Treatment × Tract: *F*(1, 31) = 5.11, *p* = 0.0309, *R*^2^ = 0.48; active tDCS: *b* = −5.18, SE = 1.42, 95% CI = (−8.06, −2.29); sham: *b* = −0.58, SE = 1.55, 95% CI = (−3.73, 2.57)]. These effects appeared to be driven by the left frontal CPT having higher FA [Treatment × Tract: *F*(1, 16) = 14.12, *p* = 0.0017, *R*^2^ = 0.74; active tDCS: *b* = 9.62, SE = 1.46, 95% CI = (6.54, 12.71); sham: *b* = 6.26, SE = 1.46, 95% CI = (3.17, 9.35)] and lower MD [Treatment × Tract: *F*(1, 16) = 20.63, *p* = 0.0003, *R*^2^ = 0.70; active tDCS: *b* = −3.37, SE = 0.55, 95% CI = (−4.53, −2.21); sham: *b* = 0.06, SE = 0.55, 95% CI = (−1.11, 1.22)]. Similarly, the left PPC effects were driven by left parietal CPT having lower MD [Treatment × Tract: *F*(1, 31) = 4.37, *p* = 0.0449, *R*^2^ = 0.52; active tDCS: *b* = 3.41, SE = 0.84, 95% CI = (−5.12, −1.69); sham: *b* = −0.83, SE = 0.90, 95% CI = (−2.65, 1.00)].

## 4 Discussion

In the present study, we asked the question of whether baseline cerebro-cerebellar white matter integrity may predict effects of cerebellar tDCS treatment coupled with computerized aphasia therapy. Our results indicate that baseline cerebro-cerebellar white matter tract integrity predicts treatment response to cerebellar tDCS in post-stroke aphasia rehabilitation. Specifically, higher FA and lower MD in descending cortico-ponto-cerebellar pathways predicted larger gains from right cerebellar tDCS paired with language therapy, with effects sustained for 2 months post-treatment. These white matter findings occurred within the context of behavioral improvements showing that cerebellar tDCS enhanced therapy effects on untrained naming (PNT), and one measure of functional communication (ASHA-FACS QDC). However, cerebellar tDCS did not enhance behavioral therapy effects for trained naming (Naming 80) and another measure of functional communication (ASHA-FACS CI). These findings replicates our prior work and is in line with others’ studies (1, 3, 30–32, 46, 70) showing that pairing cerebellar tDCS with aphasia therapy can yield durable improvements while also highlighting that the magnitude and specificity of adjuvant benefits depend on the language measure tested. For a discussion of the behavioral tDCS effects of trained and untrained tasks, we refer the reader to Sebastian et al. (33) and Kim et al. (47).

Our primary hypothesis was supported: baseline properties of cerebro-cerebellar white-matter tracts predicted or moderated the treatment effects of cerebellar tDCS relative to sham. The most consistent pattern emerged for descending cortico-ponto-cerebellar pathways connecting the lesioned left cerebral cortex to the stimulated right posterolateral cerebellum. For functional communication, the integrity of left-hemisphere descending pathways was the strongest predictor of cerebellar tDCS treatment gains. Specifically, left FPC integrity reliably predicted tDCS improvements on both ASHA-FACS scales (CI and QDC), while right MCP showed associations with untrained naming gains on the PNT. These findings underscore that the cerebellum’s capacity to enhance language recovery depends critically on preserved input from spared left-hemisphere tissue and is consistent with previous findings on the relevance of cortico-ponto-cerebellar pathways for stroke recovery and language processing (44, 67, 68, 75). Notably, distinct white matter pathways also predicted response to language therapy alone (sham condition). For example, left OPC integrity predicted untrained naming (PNT) gains in the sham group, consistent with studies showing that white matter integrity predicts responsiveness to language rehabilitation independently of neurostimulation (43, 76–78). This dissociation suggests that different cortico-ponto-cerebellar pathways may support distinct mechanisms of language recovery.

The trained naming task (Naming 80) showed a different predictive pattern: bilateral pathways were associated with treatment gains, including right FPC, right PPC, and right OPC, tracts connecting the right cerebral cortex to the left (non-stimulated) cerebellum. This divergence likely reflects differences in the neural substrates supporting specific treatment effects versus generalization (78, 79). Trained naming involves repeated practice on a constrained set of items, which may engage more distributed or bilateral cerebellar networks related to learning. Prior neuroimaging work demonstrates that cognitively demanding tasks engage bilateral cerebellar lobules along with prefrontal and parietal cortices (21), supporting this interpretation. The bilateral pattern for trained items suggests that even unilateral cerebellar stimulation can modulate language functions through distributed network effects.

Regarding the ascending DRTC pathway linking the stimulated right posterolateral cerebellum to the lesioned left cerebral cortex, most effects were null, with two exceptions. For functional communication ASHA-FACS CI, higher MD showed a trend-level association with greater gains after tDCS, and this pattern differed significantly from sham, where higher MD was associated with smaller gains. For the trained naming task (Naming 80), higher MD predicted greater gains in the sham condition only. Given that the DRTC is the primary ascending pathway conveying cerebellar output to cortical association areas, and that our prior work demonstrated positive associations between DRTC integrity and naming performance in chronic post-stroke aphasia (44), it was surprising that we did not find more associations with language gains after cerebellar tDCS. This may suggest that descending cortico-cerebellar input is more critical than ascending cerebellar output for determining cerebellar tDCS response. Alternatively, it is possible that sample size limited our ability to detect more subtle modulation effects in this pathway.

The secondary hypothesis that cathodal cerebellar tDCS may be more likely to show treatment and predictive effects was not supported. Contrary to our hypothesis, but consistent with several prior reports (30–33), the polarity effects (anode versus cathode) were mixed across the outcome measures, and predictive relations with baseline tract integrity were largely similar for anodal and cathodal stimulation. Multiple factors likely contribute to polarity inconsistency in the cerebellum (80–83) including neuroanatomical factors interacting with the tDCS stimulation, such as the complexity of gyral folding, the types of neuronal cells engaged, and other individual differences. Therefore, the potential impact of tDCS polarity on treatment efficacy and predictive effects in the specific context of cerebellar tDCS combined with aphasia therapy remains an open question for future research.

A highly consistent pattern across tasks and the descending tracts was that larger cerebellar tDCS treatment gains were associated with a diffusivity profile indicative of higher white-matter integrity [higher FA, lower MD (77, 78, 102)], particularly within the context of stroke-induced white matter degeneration (75, 84, 85). Participants with lower baseline integrity in the descending cortico-ponto-cerebellar pathways tended to show smaller cerebellar tDCS gains, underscoring that the cerebellum’s capacity to influence language depends on the inputs from spared left-hemisphere tissue. This is consistent with anterograde degeneration following cortical lesions (75) and with reports that have shown that white matter alterations in the cerebellum, the superior and middle cerebellar peduncles, and the pons correlate with recovery of motor function after stroke (80, 86). However, it is also important to acknowledge the known challenges of interpreting microstructural tissue integrity from DTI-derived metrics, especially in relatively low-resolution diffusion data (87–89, 102). Nevertheless, the convergent association between better-organized white matter and stronger cerebellar tDCS response parallels broader neuromodulation findings (42, 43, 76, 90, 91), i.e., individuals with relatively preserved structural connectivity within functionally relevant networks and/or adaptive reorganization of spared regions are more likely to benefit (44, 81), in keeping with state-dependent and metaplastic accounts of tDCS (92).

Because we created the cerebro-cerebellar tracts by combining the cerebellar peduncles with the contralateral corticopontine projections, we anticipated that the predictive effects may be driven by either or both of the separate segments. Across composite tracts, language measures, DTI metrics, and tDCS polarity groups, both corticopontine and cerebellar segments contributed relatively equally to predictive effects. For the cerebellar peduncles specifically, predictive effects involved both the stimulated (right) and non-stimulated (left) hemispheres. Notably, a functional dissociation emerged between cerebellar peduncle types: MCP integrity predicted treatment gains across all language measures, whereas SCP integrity predicted only functional communication outcomes (ASHA-FACS CI and QDC). This pattern suggests selective associations between specific cerebellar white matter pathways and language functions. The measures themselves differ meaningfully: ASHA-FACS provides global ratings of functional communication based on clinician and caregiver observations, whereas PNT and Naming80 are objective, performance-based measures of discrete naming ability. This distinction may be critical for understanding the dissociation. The MCP, as part of the afferent pathway, transmits task-specific information from cortex to cerebellum and supports lower-level language functions. In contrast, the SCP, as part of the efferent pathway, conveys cerebellar output back to cortex and supports higher-level language functions (64, 67, 82). Our differential findings may therefore reflect this functional dissociation between cerebellar peduncles, consistent with other evidence of pathway-specific contributions to distinct language processes (45, 66).

The bilaterality of predictive effects from the cerebro-cerebellar tracts or tract segments (e.g., peduncles) was surprising given that the cerebellar stimulation was lateralized (right hemisphere), the cerebro-cerebellar tracts are contralateral, and the cerebellar peduncles do not bridge within the cerebellum. There are several possible explanations to this finding. First, although our montage concentrates the electric field in the right cerebellum, current field modeling indicates some spread to the left cerebellum (32). Second, indirect modulation of the non-targeted cerebellum is plausible via propagation of tDCS effects through structural connections directly between the cerebellar hemispheres (e.g., cerebellar commissure) or indirectly via cross-hemispheric connections between cortices (e.g., corpus callosum) and/or via feedback loops or interactions between cerebro-cerebellar tracts. In either case, indirect modulation of the non-targeted cerebellum may have contributed to its predictive effects. Functional neuroimaging studies provide convergent evidence for such distant effects of cerebellar tDCS, including modulation of BOLD activity and connectivity in contralateral cortical nodes within motor and cognitive networks and changes in language-related circuits (52, 83). Together, these observations suggest that cerebellar tDCS acts as a network-level intervention whose behavioral impact depends on bilateral cerebro-cerebellar loop integrity.

An important point to note is that we did not detect any significant cerebellar tDCS carryover effects (see section 2.8.1), which seems likely because we took several steps to minimize the potential for carryover effects. We used a 2-month break in between phase 1 and phase 2 with the assumption that the tDCS effect will have washed out by that time. In addition, we also adopted an analysis approach utilized by our colleagues (93, 94) to allow for the case that the tDCS effect does not wash out in 2 months. In our analysis, we used the “change in naming or functional communication” as the main outcome, i.e., the score at each post-treatment/follow-up minus the score at baseline of the respective phase. This means that any effect of tDCS versus sham found in phase 2 will not reflect the improvement itself at the level of performance in naming, i.e., the fact that the participants who got tDCS first are naming more accurately than those who got sham. Therefore, any effect of tDCS versus sham that carries over from 2 months of phase 1 to only the level of performance in phase 2 will cancel in the tDCS versus sham comparisons of “change in naming” at phase 2. In this way, we eliminated one of the possible “carryover” effects of tDCS: the improvement at the mere level of performance (94).

This study has several limitations. First, while our sample size is comparable to other cerebellar tDCS studies, it constrained statistical power and precluded more complex modeling approaches, such as including random effects for treatment or timepoint and testing higher-order interactions (e.g., polarity-by-tract-by-time effects). Second, our diffusion acquisition parameters, though clinically pragmatic, were limited in diffusion weighting, gradient count, and voxel size. These constraints precluded advanced preprocessing techniques and reliable decussation estimation, necessitating manual combination of cerebral and cerebellar tract segments. As with all DTI-based studies, our microstructural inferences remain indirect and sensitive to acquisition and modeling choices. Third, we did not differentiate between aphasia subtypes (fluent versus non-fluent) and stroke types (ischemic vs. hemorrhagic), which may influence outcomes (95). However, we note that our treatment targeted naming, a core deficit across aphasia subtypes, and our sample consisted exclusively of chronic stroke patients (>6 months), a phase when acute pathophysiological differences between stroke types have largely resolved. Fourth, we did not include canonical perisylvian language tracts (e.g., arcuate fasciculus, superior longitudinal fasciculus, inferior fronto-occipital fasciculus) as comparison structures. Our focus on cerebro-cerebellar pathways was driven by our research question examining cerebellar stimulation effects; however, without these control tracts, we cannot definitively determine whether our predictive effects are specific to cerebellar circuitry or reflect general language-related white matter integrity. Prior literature shows that damage to these tracts robustly predicts aphasia severity and therapy response (96–99). A critical next step is therefore to test whether cerebellar tract integrity explains unique variance in tDCS response beyond lesion burden and perisylvian tract status, and whether combining these biomarkers improves patient stratification.

In conclusion, this exploratory study provides novel evidence that baseline cerebro-cerebellar tract integrity moderates the efficacy of right cerebellar tDCS delivered in conjunction with aphasia therapy: higher FA and lower MD in these loops predict larger short- and long-term gains on naming and functional communication. The bilateral nature of predictive effects, despite unilateral stimulation, is consistent with network-level propagation documented in functional neuroimaging studies of cerebellar tDCS. Future work should (a) replicate these findings in larger, more diverse cohorts; (b) incorporate canonical language tracts and lesion metrics to test specificity; (c) use advanced diffusion and functional imaging to refine tractography and quantify network-level modulation; and (d) leverage individualized modeling and state-dependent protocols to optimize polarity, dosing, and montage. Together, these steps will enable biomarker-guided, mechanistically informed personalization of adjuvant neuromodulation for post-stroke aphasia.

## Data Availability

The raw data supporting the conclusion of this article will be made available by the authors, without undue reservation.
