# Supplementary material for: White matter predictors of cerebellar tDCS treatment effects in aphasia rehabilitation
Source: Front Neurol. 2026 Feb 20;17:1659337. doi: 10.3389/fneur.2026.1659337 (PMC12962960; doi:10.3389/fneur.2026.1659337)
Supplement: Supplementary file 1 [file Data_Sheet_1.pdf]

Supplemental Materials (Aphasia Cerebellar tDCS and DTI)

Appendix 1

Figure S1. Participant flow diagram. Tx: Treatment. Ax: Assessment. 2W: 2 weeks. 2M: 2 months. Note, all analyses and results reported in this publication are based on a final study sample (n = 19) of participants, with baseline MRI randomly assigned to either cathodal tDCS polarity (n = 10) or anodal tDCS polarity (n = 9), which is only a subset of the larger sample size in the ongoing study reported in the figure below.

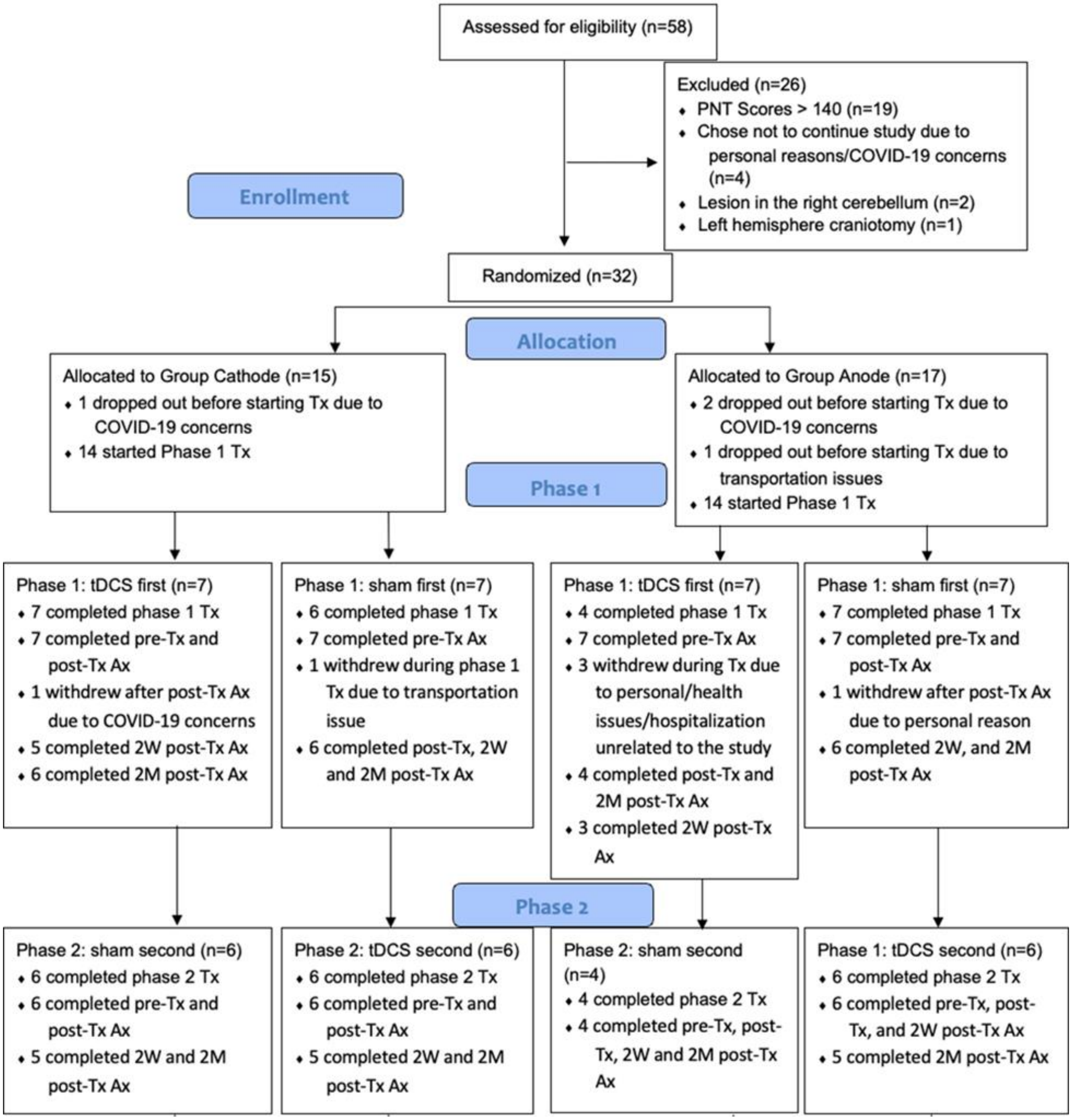

Appendix 2A

Behavioral Results: Treatment effects on language outcomes (tDCS groups combined)

Naming 80 (Trained)

tDCS Polarity Groups Combined. The Treatment x Timepoint interaction was not significant, $F(2, 97) = 0.02$, $p = 0.9825$, $R2 = 0.20$). There was a reliable increase in accuracy at each post-treatment timepoint for active tDCS (post-tx: $\Delta M = 57.00\%$, SE = 12.90%, 95% CI = [31.40%, 82.60%]; 2-wks post-tx: $\Delta M = 53.20\%$, SE = 13.20%, 95% CI = [27.00%, 79.50%]; 2-mos post-tx: $\Delta M = 42.30\%$, SE = 13.20%, 95% CI = [16.10%, 68.60%]) as well as for sham tDCS (post-tx: $\Delta M = 64.70\%$, SE = 13.20%, 95% CI = [38.50%, 91.00%]; 2-wks post-tx: $\Delta M = 56.10\%$, SE = 13.60%, 95% CI = [29.10%, 83.20%]; 2-mos post-tx: $\Delta M = 48.80\%$, SE = 13.60%, 95% CI = [21.70%, 75.90%]), resulting in non-significant differences (active – sham) at each timepoint (post-tx: $t(97) = -0.42$, $p = 0.6768$, $d = -0.14$, 95% CI = [-0.79, 0.52]); 2-wks post-tx: $t(97) = -0.15$, $p = 0.8784$, $d = -0.05$, 95% CI = [-0.72, 0.62]); 2-mos post-tx: $t(97) = -0.34$, $p = 0.7342$, $d = -0.12$, 95% CI = [-0.79, 0.56]). The main effect of Treatment, when averaging across timepoints, was also not significant, $F(1, 99) = 0.28$, $p = 0.5985$, $R2 = 0.20$, $d = -0.10$, 95% CI = [-0.49, 0.28]). There was a reliable increase in accuracy for active tDCS ($\Delta M = 50.80\%$, SE = 7.58%, 95% CI = [35.80%, 65.90%]) and sham ($\Delta M = 56.60\%$, SE = 7.83%, 95% CI = [41.00%, 72.10%]).

PNT (Untrained)

tDCS Polarity Groups Combined. The Treatment x Timepoint interaction was not significant, $F(2, 96) = 0.79$, $p = 0.4530$, $R2 = 0.07$). There were reliable increases in accuracy for most timepoints for active tDCS (post-tx: $\Delta M = 26.21\%$, SE = 11.00%, 95% CI = [4.45%, 48.00%]; 2-wks post-tx: $\Delta M = 20.09\%$, SE = 11.30%, 95% CI = [-2.26%, 42.40%]; 2-mos post-tx: $\Delta M = 40.18\%$, SE = 11.30%, 95% CI = [17.84%, 62.50%]) but not for sham tDCS (post-tx: $\Delta M = 14.46\%$, SE = 11.30%, 95% CI = [-7.88%, 36.90%]; 2-wks post-tx: $\Delta M = 4.71\%$, SE = 11.90%, 95% CI = [-19.01%, 28.40%]; 2-mos post-tx: $\Delta M = 1.97\%$, SE = 11.60%, 95% CI = [-21.05%, 25.00%]), resulting in non-significant differences (active – sham) for post-tx ($t(96) = 0.75$, $p = 0.4564$, $d = 0.25$, 95% CI = [-0.41, 0.90]) and 2-wks post-tx ($t(96) = 0.94$, $p = 0.3506$, $d = 0.32$, 95% CI = [-0.36, 1.01]) but the difference at 2-mos post-tx was significant with a large effect size ($t(96) = 2.37$, $p = 0.0199$, $d = 0.80$, 95% CI = [0.12, 1.48]). The main effect of Treatment, when averaging across timepoints, was marginally significant with a medium effect size, $F(1, 98) = 5.37$, $p = 0.0225$, $R2 = 0.06$, $d = 0.45$, 95% CI = [0.06, 0.84]. There was a reliable increase in accuracy for active tDCS ($\Delta M = 28.74\%$, SE = 6.49%, 95% CI = [15.85%, 41.60%]) but not sham ($\Delta M = 7.08\%$, SE = 6.77%, 95% CI = [-6.35%, 20.50%]).

ASHA-FACS (CI Scale)

tDCS Polarity Groups Combined. The Treatment x Timepoint interaction was not significant, $F(2, 94) = 1.16$, $p = 0.3168$, $R2 = 0.07$). There were reliable increases in accuracy at all timepoints for active tDCS (post-tx: $\Delta M = 28.70\%$, SE = 6.13%, 95% CI = [16.56%, 40.90%]; 2-wks post-tx: $\Delta M = 28.00\%$, SE = 6.71%, 95% CI = [14.66%, 41.30%]; 2-mos post-tx: $\Delta M = 22.60\%$, SE = 6.47%, 95% CI = [9.71%, 35.40%]) and also for sham tDCS (post-tx: $\Delta M = 16.10\%$, SE = 6.29%, 95% CI = [3.56%, 28.60%]; 2-wks post-tx: $\Delta M = 28.70\%$, SE = 6.48%, 95% CI = [15.83%, 41.60%]; 2-mos post-tx: $\Delta M = 28.60\%$, SE = 6.48%, 95% CI = [15.70%, 41.50%]), resulting in non-significant differences (active – sham) at all timepoints (post-tx: $t(94) = 1.44$, $p = 0.1526$, $d = 0.48$, 95% CI = [-0.18, 1.13]; 2-wks post-tx: $t(94) = -0.08$, $p = 0.9378$, $d = -0.03$, 95% CI = [-0.72, 0.67]; 2-mos post-tx: ($t(94) = -0.66$, $p = 0.5123$, $d = -0.23$, 95% CI = [-0.91, 0.46]). The main effect of Treatment, when averaging across timepoints, was also not significant ($F(1, 96) = 0.19$, $p = 0.6651$, $R2 = 0.05$, $d = 0.09$, 95% CI = [-0.31, 0.48]). There was a reliable increase in accuracy for active tDCS ($\Delta M = 26.60\%$, SE = 3.76%, 95% CI = [19.20%, 34.10%]) which was similar to sham ($\Delta M = 24.30\%$, SE = 3.76%, 95% CI = [16.90%, 31.80%]).

ASHA-FACS (QDC Scale)

tDCS Polarity Groups Combined. The Treatment x Timepoint interaction was not significant, $F(2, 94) = 0.18$, $p = 0.8354$, $R2 = 0.24$). There were reliable increases in accuracy at all timepoints for active tDCS (post-tx: $\Delta M = 31.81\%$, SE = 6.04%, 95% CI = [19.82%, 43.80%]; 2-wks post-tx: $\Delta M = 32.46\%$, SE = 6.61%, 95% CI = [19.33%, 45.60%]; 2-mos post-tx: $\Delta M = 30.47\%$, SE = 6.37%, 95% CI = [17.82%, 43.10%]) but not for sham (post-tx: $\Delta M = 7.93\%$, SE = 6.20%, 95% CI = [-4.38%, 20.20%]; 2-wks post-tx: $\Delta M = 12.67\%$, SE = 6.39%, 95% CI = [-0.02%, 25.30%]; 2-mos post-tx: $\Delta M = 14.07\%$, SE = 6.39%, 95% CI = [0.01%, 26.80%]), resulting in significant differences (active – sham) with large effect sizes at post-tx ($t(94) = 2.76$, $p = 0.0070$, $d = 0.91$, 95% CI = [0.24, 1.58]) and at 2-wks post-tx ($t(94) = 2.16$, $p = 0.0332$, $d = 0.75$, 95% CI = [0.05, 0.1.45]) but only marginally significant at 2-mos post-tx: ($t(94) = 1.82$, $p = 0.0721$, $d = 0.62$, 95% CI = [-0.06, 1.31]). The main effect of Treatment, when averaging across timepoints, was highly significant ($F(1, 96) = 15.18$, $p = 0.0002$, $R2 = 0.24$, $d = 0.77$, 95% CI = [0.36, 1.17]). There was a reliable increase in accuracy for active tDCS ($\Delta M = 31.70\%$, SE = 3.67%, 95% CI = [24.36%, 38.90%]) which was larger than sham ($\Delta M = 11.50\%$, SE = 3.67%, 95% CI = [4.23%, 18.80%]).

Appendix 2B

Behavioral Results: Treatment effects on language outcomes (by tDCS group)

Naming 80 (PNT Trained)

DV	IV	Group	Interaction					Timepoint	Active				Sham				Active vs Sham						
			df1	df2	F	p	R^2		ΔM	SE	CI.low	CI.high	ΔM	SE	CI.low	CI.high	ΔM	SE	t	p	d	d.low	d.high
Naming 80 (PNT Trained)	Treatment	Anode	1	46	12.74	0.0009	0.51	ALL	61.40%	6.80%	47.80%	75.10%	95.70%	6.80%	82.00%	109.40%	-34.20%	9.59%	-3.57	0.0009	-0.97	-1.56	-0.39
		Cathode	1	45	2.82	0.1001	0.30	ALL	40.70%	10.20%	20.22%	61.20%	15.80%	10.90%	-6.08%	37.80%	24.90%	14.80%	1.68	0.1001	0.46	-0.1	1.03
	Treatment x Tpt	Anode	2	44	1.48	0.2387	0.54	post1	68.10%	11.40%	45.00%	91.20%	101.60%	11.40%	78.60%	124.70%	-33.50%	16.20%	-2.08	0.0439	-0.98	-1.95	-0.01
								post2	71.40%	11.40%	48.30%	94.40%	86.20%	11.40%	63.20%	109.30%	-14.90%	16.20%	-0.92	0.3619	-0.43	-1.39	0.52
								post3	44.90%	11.40%	21.80%	67.90%	99.10%	11.40%	76.00%	122.20%	-54.20%	16.20%	-3.35	0.0016	-1.58	-2.59	-0.57
		Cathode	2	43	0.63	0.5359	0.32	post1	43.24%	16.90%	9.25%	77.20%	30.09%	17.70%	-5.63%	65.80%	13.10%	24.50%	0.54	0.5942	0.25	-0.69	1.18
								post2	37.40%	17.70%	1.68%	73.10%	23.43%	18.90%	-14.59%	61.50%	14.00%	25.80%	0.54	0.5906	0.26	-0.72	1.25
								post3	42.06%	17.70%	6.34%	77.80%	-6.66%	18.90%	-44.68%	31.40%	48.70%	25.80%	1.89	0.0654	0.92	-0.08	1.92

Separate tDCS Polarity Groups. For the anodal group, the Treatment x Timepoint interaction was not significant (F(2, 44) = 1.48, p = 0.2387, R2 = 0.54), due to reliable increases in accuracy at all timepoints for both active tDCS and sham (see post hoc tests in Appendix 1A of Supplemental Materials). However, the main effect of Treatment averaging across timepoints was significant with a large effect size, F(1, 46) = 12.74, p = 0.0009, R2 = 0.51, d = -0.97, 95% CI = [-1.56, -0.39]). There was a reliable accuracy increase for active tDCS (ΔM = 61.40%, SE = 6.80%, 95% CI = [47.80%, 75.10%]) and even larger for sham (ΔM = 95.70%, SE = 6.80%, 95% CI = [82.00%, 109.40%]).

For the cathodal group, the Treatment x Timepoint interaction was not significant (F(2, 43) = 0.63, p = 0.5359, R2 = 0.32). Accuracy increased at all timepoints for active tDCS but these changes were not significantly different from sham (see post hoc tests in Appendix 1A of Supplemental Materials). The main effect of Treatment averaging across timepoints was also not significant, F(1, 45) = 2.82, p = 0.1001, R2 = 0.30, d = 0.46, 95% CI = [-0.10, 1.03]). There was a reliable accuracy increase for active tDCS (ΔM = 40.70%, SE = 10.20%, 95% CI = [20.22%, 61.20%]) but not for sham (ΔM = 15.80%, SE = 10.90%, 95% CI = [-6.08%, 37.80%]).

PNT (Untrained)

DV	IV	Group	Interaction					Timepoint	Active				Sham				Active vs Sham						
			df1	df2	F	p	R^2		ΔM	SE	CI.low	CI.high	ΔM	SE	CI.low	CI.high	ΔM	SE	t	p	d	d.low	d.high
PNT (Untrained)	Treatment	Anode	1	46	3.29	0.0764	0.15	ALL	31.13%	9.76%	11.50%	50.80%	6.18%	9.76%	-13.50%	25.80%	24.90%	13.80%	1.81	0.0764	0.49	-0.06	1.05
		Cathode	1	44	2.29	0.1372	0.12	ALL	26.11%	7.68%	10.64%	41.60%	8.99%	8.39%	-7.92%	25.90%	17.10%	11.30%	1.51	0.1372	0.42	-0.15	0.99
	Treatment x Tpt	Anode	2	44	0.67	0.5173	0.17	post1	24.66%	16.70%	-8.93%	58.30%	18.70%	16.70%	-14.89%	52.30%	5.96%	23.50%	0.25	0.8013	0.12	-0.83	1.07
								post2	17.89%	16.70%	-15.70%	51.50%	-6.51%	16.70%	-40.11%	27.10%	24.40%	23.50%	1.04	0.3057	0.49	-0.47	1.44
								post3	50.82%	16.70%	17.23%	84.40%	6.35%	16.70%	-27.24%	39.90%	44.47%	23.50%	1.89	0.0655	0.89	-0.08	1.86
		Cathode	2	42	0.50	0.6079	0.14	post1	24.70%	12.80%	-1.05%	50.40%	11.22%	13.40%	-15.85%	38.30%	13.48%	18.60%	0.73	0.4717	0.34	-0.6	1.27
								post2	23.29%	13.40%	-3.78%	50.40%	18.24%	15.30%	-12.56%	49.00%	5.05%	20.30%	0.25	0.8045	0.13	-0.89	1.15
								post3	30.54%	13.40%	3.47%	57.60%	-1.86%	14.30%	-30.67%	26.90%	32.40%	19.50%	1.66	0.1045	0.81	-0.19	1.8

Separate tDCS Polarity Groups. For the anodal group, the Treatment x Timepoint interaction was not significant (F(2, 44) = 0.67, p = 0.5173, R2 = 0.17), due to unreliable increases in accuracy for most timepoints for both active tDCS and sham (see post hoc tests in Appendix 1A of Supplemental Materials). The main effect of Treatment averaging across timepoints was also not significant, F(1, 46) = 3.29, p = 0.0764, R2 = 0.15, d = 0.49, 95% CI = [-0.06, 1.05]). There was a reliable accuracy increase for active tDCS (ΔM =31.13%, SE = 9.76%, 95% CI = [11.50%, 50.80%]) but not for sham (ΔM = 6.18%, SE = 9.76%, 95% CI = [-13.50%, 25.80%]).

For the cathodal group, the Treatment x Timepoint interaction was not significant (F(2, 42) = 0.50, p = 0.6079, R2 = 0.14), due to unreliable increases in accuracy for most timepoints for both active tDCS and sham (see post hoc tests in Appendix 1A of Supplemental Materials). The main effect of Treatment averaging across timepoints was also not significant, F(1, 44) = 2.29, p = 0.1372, R2 = 0.12, d = 0.42, 95% CI = [-0.15, 0.99]). There was a reliable accuracy increase for active tDCS (ΔM = 26.11%, SE = 7.68%, 95% CI = [10.64%, 41.60%]) but not for sham (ΔM = 8.99%, SE = 8.39%, 95% CI = [-7.92%, 25.90%]).

ASHAFACS (CI)

DV	IV	Group	Interaction					Timepoint	Active				Sham				Active vs Sham						
			df1	df2	F	p	R^2		ΔM	SE	CI.low	CI.high	ΔM	SE	CI.low	CI.high	ΔM	SE	t	p	d	d.low	d.high
ASHAFACS (CI)	Treatment	Anode	1	44	2.69	0.1088	0.35	ALL	32.70%	4.25%	24.20%	41.30%	23.10%	4.09%	14.90%	31.40%	9.63%	5.88%	1.64	0.1088	0.46	-0.11	1.02
		Cathode	1	44	0.46	0.5005	0.06	ALL	21.20%	5.47%	10.10%	32.20%	26.50%	5.72%	15.00%	38.00%	-5.32%	7.83%	-0.68	0.5005	-0.19	-0.75	0.37
	Treatment x Tpt	Anode	2	42	0.79	0.4594	0.36	post1	31.20%	6.96%	17.20%	45.30%	15.10%	6.96%	1.10%	29.20%	16.08%	9.83%	1.64	0.1093	0.77	-0.19	1.74
								post2	39.80%	7.41%	24.90%	54.80%	26.80%	6.96%	12.70%	40.80%	13.08%	10.10%	1.29	0.2041	0.63	-0.36	1.62
								post3	26.80%	7.38%	11.90%	41.70%	27.50%	6.96%	13.50%	41.50%	-0.72%	10.20%	-0.07	0.9441	-0.03	-1.02	0.95
		Cathode	2	42	1.02	0.3705	0.09	post1	27.20%	8.77%	9.53%	44.90%	18.10%	9.22%	-0.54%	36.70%	9.15%	12.80%	0.72	0.4771	0.33	-0.6	1.27
								post2	16.80%	9.86%	-31.36%	36.70%	31.60%	9.82%	11.78%	51.40%	-14.83%	13.80%	-1.07	0.2889	-0.54	-1.55	0.48
								post3	18.20%	9.22%	-0.01%	36.80%	30.50%	9.82%	10.68%	50.30%	-12.34%	13.40%	-0.92	0.3630	-0.45	-1.43	0.54

Separate tDCS Polarity Groups. For the anodal group, the Treatment x Timepoint interaction was not significant (F(2, 42) = 0.79, p = 0.4594, R2 = 0.36), due to reliable increases in accuracy at all timepoints for both active tDCS and sham (see post hoc tests in Appendix 1A of Supplemental Materials). The main effect of Treatment averaging across timepoints was also not significant, F(1, 44) = 2.69, p = 0.1088, R2 = 0.35, d = 0.46, 95% CI = [-0.11, 1.02]). There was a reliable accuracy increase for active tDCS (ΔM =32.70%, SE = 4.25%, 95% CI = [24.20%, 41.30%]) which was similar to sham (ΔM = 23.10%, SE = 4.09%, 95% CI = [14.90%, 31.40%]).

For the cathodal group, the Treatment x Timepoint interaction was not significant (F(2, 42) = 1.02, p = 0.3705, R2 = 0.09), due to a mix of reliable or unreliable changes for both active tDCS and sham (see post hoc tests in Appendix 1A of Supplemental Materials). The main effect of Treatment averaging across timepoints was also not significant, F(1, 44) = 0.46, p = 0.5005, R2 = 0.06, d = -0.19, 95% CI = [-0.75, 0.37]). There was a reliable accuracy increase for active tDCS (ΔM = 21.20%, SE = 5.47%, 95% CI = [10.10%, 32.20%]) which was similar to sham (ΔM = 26.50%, SE = 5.72%, 95% CI = [15.00%, 38.00%]).

ASHAFACS (QDC)

DV	IV	Group	Interaction					Timepoint	Active				Sham				Active vs Sham						
			df1	df2	F	p	R^2		ΔM	SE	CI.low	CI.high	ΔM	SE	CI.low	CI.high	ΔM	SE	t	p	d	d.low	d.high
ASHAFACS (QDC)	Treatment	Anode	1	44	4.50	0.0396	0.30	ALL	32.20%	5.78%	20.55%	43.80%	15.20%	5.56%	4.03%	26.40%	17.00%	8.00%	2.12	0.0396	0.59	0.02	1.16
	Treatment	Cathode	1	44	16.65	0.0002	0.38	ALL	31.94%	3.84%	24.21%	39.70%	9.53%	4.01%	1.45%	17.60%	22.40%	5.49%	4.08	0.0002	1.14	0.52	1.75
	Treatment x Tpt	Anode	2	42	0.23	0.7958	0.30	post1	31.50%	9.56%	12.21%	50.80%	9.12%	9.56%	-10.17%	28.40%	22.38%	13.50%	1.66	0.1048	0.78	-0.19	1.75
								post2	35.60%	10.20%	15.06%	56.10%	16.99%	9.56%	-2.31%	36.30%	18.61%	13.90%	1.34	0.1889	0.65	-0.34	1.64
								post3	29.17%	10.10%	8.70%	49.60%	19.65%	9.56%	0.35%	38.90%	9.53%	13.90%	0.68	0.4984	0.33	-0.65	1.32
	Treatment x Tpt	Cathode	2	42	0.01	0.9897	0.38	post1	31.39%	6.27%	18.73%	44.10%	8.13%	6.60%	-5.18%	21.40%	23.30%	9.12%	2.55	0.0145	1.18	0.21	2.15
								post2	31.59%	7.05%	17.35%	45.80%	10.25%	7.02%	-3.92%	24.40%	21.30%	9.87%	2.156	0.0365	1.08	0.04	2.12
								post3	32.73%	6.60%	19.42%	46.00%	10.25%	7.02%	-3.92%	24.40%	22.50%	9.60%	2.34	0.0240	1.14	0.13	2.15

Separate tDCS Polarity Groups. For the anodal group, the Treatment x Timepoint interaction was not significant (F(2, 42) = 0.23, p = 0.7958, R2 = 0.30), due to reliable increases in accuracy at all timepoints for active tDCS but not for sham (see post hoc tests in Appendix 1A of Supplemental Materials). The main effect of Treatment averaging across timepoints was marginally significant with a medium effect size, F(1, 44) = 4.50, p = 0.0396, R2 = 0.30, d =

0.59, 95% CI = [0.02, 1.16]). There was a reliable accuracy increase for active tDCS (ΔM = 32.20%, SE = 5.78%, 95% CI = [20.55%, 43.80%]) which was higher than sham (ΔM = 15.20%, SE = 5.56%, 95% CI = [4.03%, 26.40%]).

For the cathodal group, the Treatment x Timepoint interaction was not significant ($F(2, 42) = 0.01$, $p = 0.9897$, $R2 = 0.38$), due to reliable increases in accuracy at all timepoints for active tDCS but not for sham (see post hoc tests in Appendix 1A of Supplemental Materials). The main effect of Treatment, averaging across timepoints, was highly significant with a large effect size, $F(1, 44) = 16.65$, $p = 0.0002$, $R2 = 0.38$, $d = 1.14$, 95% CI = [0.52, 1.75]). There was a reliable accuracy increase for active tDCS (ΔM = 31.94%, SE = 3.84%, 95% CI = [24.21%, 39.70%]) which was higher than sham (ΔM = 9.53%, SE = 4.01%, 95% CI = [1.45%, 17.60%]).

Appendix 2C

Behavioral Results: Treatment x Order effects on language outcomes (tDCS groups combined)

DV	Term	df1	df2	F	p	R^2
PNT	Tretament x Order	1	99	0.06	0.8022	0.05
Naming 80	Treatment x Order	1	100	0.01	0.9174	0.19
ASHA-FACS (CI)	Tretament x Order	1	97	0.29	0.5907	0.04
ASHA-FACS (QDC)	Treatment x Order	1	97	0.13	0.7208	0.72

Appendix 3A

Baseline tracts (FPC, OPC, PPC, DRTC) predicting language outcomes for both tDCS groups combined

PNT (Untrained)

DV	Tract	DTI	Interaction (Treatment * Tract)					Active				Sham			
			df1	df2	F	p	R^2	b	SE	CI.low	CI.high	b	SE	CI.low	CI.high
PNT Untrained	Left FPC	FA	1	49	1.46	0.2321	0.18	1.86	2.92	-4.00	7.72	-2.45	2.93	-8.33	3.43
		MD	1	49	4.11	0.0481	0.21	-2.73	2.12	-7.00	1.53	2.42	2.17	-1.94	6.78
	Right FPC	FA	1	96	0.00	0.9598	0.06	0.62	2.57	-4.48	5.72	0.44	2.63	-4.79	5.67
		MD	1	96	0.25	0.6154	0.07	-1.08	2.12	-5.29	3.13	-2.47	2.14	-6.73	1.78
	Left PPC	FA	1	77	0.73	0.3957	0.14	1.31	2.22	-3.11	5.73	3.81	2.24	-0.66	8.28
		MD	1	77	2.32	0.1315	0.14	0.24	1.93	-3.60	4.08	-3.84	2.00	-7.82	0.13
	Right PPC	FA	1	96	0.52	0.4725	0.08	1.23	3.28	-5.29	7.74	4.61	3.49	-2.32	11.54
		MD	1	96	0.00	0.9515	0.09	-3.22	2.24	-7.67	1.23	-3.41	2.24	-7.86	1.05
	Left OPC	FA	1	78	7.74	0.0068	0.19	-2.07	2.15	-6.34	2.21	6.59	2.28	2.05	11.13
		MD	1	78	7.34	0.0083	0.19	0.49	1.11	-1.73	2.70	-3.32	1.13	-5.57	-1.08
	Right OPC	FA	1	96	0.00	0.9845	0.06	0.55	4.05	-7.49	8.58	0.66	4.42	-8.11	9.43
		MD	1	96	0.43	0.5145	0.08	-2.28	1.57	-5.40	0.85	-0.86	1.59	-4.02	2.30
	Right DRTC	FA	1	89	0.00	0.9725	0.08	0.25	1.48	-2.70	3.19	0.18	1.54	-2.88	3.23
		MD	1	89	0.09	0.7597	0.08	0.02	0.55	-1.07	1.12	-0.21	0.62	-1.45	1.02
	Left DRTC	FA	1	96	0.06	0.8115	0.06	0.43	2.73	-4.99	5.86	1.37	2.90	-4.40	7.14
		MD	1	96	0.18	0.6693	0.06	-0.18	0.69	-1.54	1.18	0.25	0.74	-1.22	1.71

OPC. Baseline FA of the left OPC significantly interacted with Treatment such that higher FA predicted higher PNT improvements across all timepoints for the sham condition but not tDCS. Baseline MD of the left OPC also significantly interacted with Treatment such that lower MD predicted higher PNT improvements over time for the sham condition but not tDCS. These findings are also consistent with observed interactions between Treatment and the laterality index (LI) of the FA and MD values at baseline.

Naming 80 (PNT Trained)

DV	Tract	DTI	Interaction (Treatment * Tract)					Active				Sham			
			df1	df2	F	p	R^2	b	SE	CI.low	CI.high	b	SE	CI.low	CI.high
Naming 80 (PNT Trained)	Left FPC	FA	1	50	0.18	0.6775	0.18	7.70	3.67	0.33	15.10	5.82	3.67	-1.55	13.20
		MD	1	50	0.38	0.5420	0.37	-10.49	2.37	-15.30	-5.72	-8.74	2.37	-13.50	-3.97
	Right FPC	FA	1	97	7.44	0.0076	0.26	7.50	2.89	1.77	13.23	-3.50	2.96	-9.37	2.37
		MD	1	97	0.41	0.5259	0.21	-2.80	2.47	-7.71	2.11	0.41	2.47	-4.48	5.31
	Left PPC	FA	1	78	2.14	0.1479	0.22	5.48	2.79	-0.07	11.03	0.11	2.82	-5.51	5.73
		MD	1	78	5.30	0.0240	0.24	-4.60	2.41	-9.40	0.20	3.10	2.47	-1.82	8.02
	Right PPC	FA	1	97	9.03	0.0034	0.28	12.50	3.66	5.25	19.76	-3.18	3.89	-10.89	4.53
		MD	1	97	0.30	0.5845	0.22	-4.33	2.63	-9.54	0.88	-2.36	2.62	-7.56	2.83
	Left OPC	FA	1	79	0.34	0.5622	0.21	0.91	2.81	-4.69	6.51	-1.46	2.98	-7.40	4.48
		MD	1	79	2.71	0.1040	0.23	-1.50	1.44	-4.37	1.37	1.49	1.46	-1.40	4.39
	Right OPC	FA	1	97	8.53	0.0043	0.26	11.93	4.53	2.94	20.92	-6.89	4.92	-16.65	2.88
		MD	1	97	0.00	0.9803	0.21	-1.43	1.85	-5.09	2.23	-1.37	1.84	-5.02	2.29
	Right DRTC	FA	1	90	1.93	0.1678	0.20	1.85	1.78	-1.69	5.39	-1.56	1.85	-5.24	2.12
		MD	1	90	7.94	0.0059	0.25	-0.69	0.64	-1.96	0.58	1.87	0.72	0.43	3.31
	Left DRTC	FA	1	97	0.28	0.6012	0.20	0.04	3.18	-6.28	6.36	2.41	3.33	-4.20	9.02
		MD	1	97	0.82	0.3675	0.20	0.23	0.80	-1.35	1.81	-0.80	0.85	-2.49	0.89

FPC. Baseline FA of the right FPC significantly interacted with Treatment such that higher FA predicted higher improvements across all timepoints for tDCS but not sham. Baseline

MD of the left FPC had a significant main effect in general such that lower MD predicted higher improvements across all timepoints and both treatment conditions.

PPC. Baseline FA of the right PPC significantly interacted with Treatment such that higher FA predicted higher improvements across all timepoints for tDCS but not sham.

OPC. Baseline FA of the right OPC significantly interacted with Treatment such that higher FA predicted higher improvements across all timepoints for tDCS (b = 11.93, 95% CI [2.94, 20.92]) but not sham (b = -6.89, 95% CI [-16.65, 2.88]).

DRTC. Baseline MD of the right DRTC significantly interacted with Treatment (F(1, 90) = 7.94, p = 0.0059, R2 = 0.25) such that higher MD predicted higher improvements across all timepoints for sham (b = 1.87, 95% CI [0.43, 3.31]) but not tDCS (b = -0.69, 95% CI [-1.96, 0.58]).

ASHAFACS (CI)

DV	Tract	DTI	Interaction (Treatment * Tract)					Active				Sham			
			df1	df2	F	p	R^2	b	SE	CI.low	CI.high	b	SE	CI.low	CI.high
ASHAFACS (CI)	Left FPC	FA	1	48	10.18	0.0025	0.32	7.25	1.58	4.06	10.43	1.14	1.53	-1.94	4.22
		MD	1	48	8.48	0.0054	0.33	-5.29	1.15	-7.59	-2.99	-1.29	1.13	-3.57	0.99
	Right FPC	FA	1	94	2.53	0.1148	0.07	2.46	1.51	-0.54	5.47	-0.80	1.46	-3.69	2.09
		MD	1	94	0.93	0.3387	0.10	-2.65	1.17	-4.98	-0.33	-1.19	1.16	-3.50	1.12
	Left PPC	FA	1	75	8.04	0.0059	0.13	1.70	1.25	-0.79	4.18	-2.89	1.23	-5.35	-0.44
		MD	1	75	0.24	0.6280	0.07	0.81	1.12	-1.43	3.04	1.56	1.13	-0.69	3.80
	Right PPC	FA	1	94	6.24	0.0142	0.10	3.24	1.89	-0.51	6.98	-3.34	1.92	-7.15	0.47
		MD	1	94	4.97	0.0282	0.10	-2.83	1.25	-5.31	-0.35	0.96	1.24	-1.51	3.43
	Left OPC	FA	1	77	2.73	0.1028	0.09	1.13	1.38	-1.62	3.87	-2.11	1.42	-4.94	0.71
		MD	1	77	0.41	0.5255	0.07	0.17	0.70	-1.23	1.57	0.74	0.71	-0.67	2.15
	Right OPC	FA	1	94	0.19	0.6675	0.06	2.39	2.29	-2.15	6.93	1.00	2.46	-3.88	5.87
		MD	1	94	0.60	0.4404	0.14	-2.43	0.85	-4.12	0.73	-1.53	0.85	-3.21	0.16
	Right DRTC	FA	1	87	1.98	0.1631	0.05	-0.92	0.86	-2.63	0.80	0.74	0.89	-1.03	2.51
		MD	1	87	9.18	0.0032	0.12	0.59	0.31	-0.03	1.20	-0.74	0.35	-1.43	-0.05
	Left DRTC	FA	1	94	0.16	0.6928	0.09	2.98	1.54	-0.09	6.05	2.13	1.57	-0.99	5.24
		MD	1	94	0.25	0.6141	0.15	-1.09	0.37	-1.82	-0.35	-0.82	0.39	-1.59	-0.05

FPC. Baseline FA of the left FPC significantly interacted with Treatment (F(1, 48) = 10.17, p = 0.0025, R2 = 0.32) such that higher FA predicted higher improvements across all timepoints for tDCS (b = 7.25, 95% CI [4.06, 10.43]) but not sham (b = 1.14, 95% CI [-1.94, 4.22]). Baseline MD of the left FPC also significantly interacted with Treatment (F(1, 48) = 8,48, p = 0.0054, R2 = 0.33) such that lower MD predicted higher improvements across all timepoints for tDCS (b = -5.29, 95% CI [-7.59, -2.99]) but not sham (b = -1.29, 95% CI [-3.57, 0.99]).

OPC. Baseline MD of the right OPC had a significant main effect in general such that lower MD predicted higher improvements across all timepoints and both treatment conditions.

PPC. Baseline FA of the left PPC significantly interacted with Treatment (F(1, 75) = 8.03, p = 0.0059, R2 = 0.13) such that lower FA predicted higher improvements across all timepoints for sham (b = -2.89, 95% CI [-5.35, -0.44]) and a trend towards higher FA predicting higher improvements for tDCS (b = 1.70, 95% CI [-0.79, 4.18]). Baseline FA of the right PPC

also significantly interacted with Treatment (F(1, 94) = 6.24, p = 0.0142, R2 = 0.10) with a trend towards lower FA predicting higher improvements across all timepoints for sham (b = -3.34, 95% CI [-7.15, 0.47]) and a trend towards higher FA predicting higher improvements for tDCS (b = 3.24, 95% CI [-0.51, 6.98]).

DRTC. Baseline MD of the right DRTC significantly interacted with Treatment (F(1, 87) = 9.18, p = 0.0032, R2 = 0.12) such that lower MD predicted higher improvements across all timepoints for sham (b = -0.74, 95% CI [-1.43, -0.05]) and a trend towards higher MD predicting higher improvements for tDCS (b = 0.59, 95% CI [-0.03, 1.20]). Baseline MD of the left DRTC had a significant main effect in general such that lower MD predicted higher improvements across all timepoints and both treatment conditions.

ASHAFACS (QDC)

DV	Tract	DTI	Interaction (Treatment * Tract)					Active				Sham			
			df1	df2	F	p	R^2	b	SE	CI.low	CI.high	b	SE	CI.low	CI.high
ASHAFACS (QDC)	Left FPC	FA	1	48	12.09	0.0011	0.49	7.17	1.46	4.24	10.10	1.04	1.41	-1.79	3.88
		MD	1	48	11.46	0.0014	0.56	-6.01	0.98	-7.98	-4.05	-2.04	0.97	-3.99	-0.09
	Right FPC	FA	1	94	4.73	0.0322	0.27	2.80	1.46	-0.11	5.71	-1.51	1.41	-4.31	1.29
		MD	1	94	0.11	0.7451	0.25	1.12	1.16	-1.19	3.42	1.61	1.15	-0.68	3.90
	Left PPC	FA	1	75	0.26	0.6113	0.24	0.25	1.32	-2.38	2.87	-0.63	1.30	-3.22	1.97
		MD	1	75	1.55	0.2172	0.27	0.14	1.12	-2.10	2.38	2.06	1.13	-0.19	4.31
	Right PPC	FA	1	94	2.47	0.1198	0.25	2.13	1.87	-1.59	5.84	-1.98	1.91	-5.76	1.80
		MD	1	94	0.26	0.6138	0.25	1.39	1.25	-1.09	3.87	0.53	1.24	-1.93	2.99
	Left OPC	FA	1	77	3.06	0.0840	0.35	-3.51	1.21	-5.92	-1.09	-0.48	1.25	-2.97	2.00
		MD	1	77	0.53	0.4696	0.36	1.72	0.61	0.51	2.93	1.16	0.61	-0.06	2.38
	Right OPC	FA	1	94	4.13	0.0448	0.28	0.81	2.19	-3.53	5.15	-5.46	2.35	-10.12	-0.80
		MD	1	94	1.01	0.3186	0.24	-0.73	0.89	-2.45	1.00	0.46	0.86	-1.26	2.17
	Right DRTC	FA	1	87	0.02	0.8794	0.26	-0.49	0.83	-2.14	1.16	-0.32	0.86	-2.02	1.38
		MD	1	87	0.00	0.9598	0.26	0.20	0.31	-0.42	0.81	0.22	0.34	-0.47	0.90
	Left DRTC	FA	1	94	0.68	0.4104	0.24	-1.20	1.54	-4.26	1.86	0.57	1.56	-2.53	3.68
		MD	1	94	0.00	0.9925	0.24	0.09	0.38	-0.67	0.85	0.09	0.40	-0.71	0.88

FPC. Baseline FA of the left FPC significantly interacted with Treatment (F(1, 48) = 12.09, p = 0.0011, R2 = 0.49) such that higher FA predicted higher improvements across all timepoints for tDCS (b = 7.17, 95% CI [4.24, 10.10]) but not sham (b = 1.04, 95% CI [-1.79, 3.88]). Baseline MD of the left FPC also significantly interacted with Treatment (F(1, 48) = 11.46, p = 0.0014, R2 = 0.56) such that lower MD predicted higher improvements across all timepoints for tDCS (b = -6.01, 95% CI [-7.98, -4.05]) and less so for sham (b = -2.04, 95% CI [-3.99, -0.09]).

OPC. Baseline MD of the right OPC had a significant main effect in general such that higher MD predicted higher improvements across all timepoints and both treatment conditions.

Appendix 3B

Baseline tracts (bilateral FPC, OPC, PPC, DRTC) predicting language outcomes for separate tDCS groups

PNT (Untrained)

Anodal tDCS. Baseline MD of the left PPC significantly interacted with Treatment such that higher MD predicted higher improvements across all timepoints for tDCS but not sham. Left OPC significantly interacted with Treatment such that lower FA and higher MD trend predicted tDCS improvements but higher FA and lower MD predicted sham improvements. Baseline MD of right DRTC also interacted with Treatment such that there was a trend for lower MD to predict more improvements for sham but not tDCS.

Finally, there was a three-way interaction between baseline MD of the right FPC and both Treatment and Timepoint. Within the tDCS condition, there was a significant interaction with Timepoint such that lower MD was associated with higher improvements after two weeks but higher MD was associated with higher improvements after treatment and after two months. Within the sham condition, there was no significant interaction with Timepoint and baseline MD did not reliably predict improvements at any of the timepoints.

tDCS Group (Anode)															
DV	Tract	DTI	nteraction (Treatment * Tract					Active				Sham			
			df1	df2	F	p	R^2	b	SE	CI.low	CI.high	b	SE	CI.low	CI.high
PNT (Untrained)	Left FPC	FA	1	26	0.03	0.8586	###	−1.342	4.37	−10.33	7.65	−0.289	4.37	−9.280	8.70
		MD	1	26	0.01	0.9307	###	1.57	5.32	−9.350	12.50	1.32	5.32	−9.600	12.20
	Right FPC	FA	1	44	2.38	0.1304	###	−4.199	3.78	−11.82	3.42	3.89	3.78	−3.730	11.51
		MD	1	44	2.46	0.1243	###	1.98	3.54	−5.160	9.11	−5.786	3.54	−12.92	1.35
	Left PPC	FA	1	38	5.78	0.0212	0.28	−7.089	3.85	−14.89	0.71	5.06	3.85	−2.740	12.86
		MD	1	38	10.23	0.0028	0.34	6.77	3.13	0.44	13.10	−2.847	3.13	−9.181	3.49
	Right PPC	FA	1	44	5.06	0.0295	0.22	−7.843	5.02	−17.96	2.27	7.93	5.02	−2.190	18.04
		MD	1	44	0.04	0.8362	0.19	−5.099	5.31	−15.80	5.59	−6.567	5.31	−17.30	4.12
	Left OPC	FA	1	32	7.10	0.0120	0.32	−4.670	3.17	−11.122	1.78	6.62	3.17	0.17	13.07
		MD	1	32	9.25	0.0047	0.35	1.93	1.57	−1.270	5.13	−2.960	1.57	−6.160	0.24
	Right OPC	FA	1	44	0.56	0.4594	0.16	−0.852	6.20	−13.340	11.60	5.41	6.20	−7.080	17.90
		MD	1	44	1.15	0.2897	0.19	−3.820	2.53	−8.930	1.29	−0.423	2.53	−5.530	4.68
	Right DRTC	FA	1	38	3.21	0.0814	0.24	−4.399	3.55	−11.59	2.79	4.15	3.55	−3.04	11.35
		MD	1	38	7.63	0.0088	0.32	−2.888	5.10	−13.20	7.43	−9.530	5.10	−19.90	0.79
	Left DRTC	FA	1	44	1.55	0.2196	0.18	−1.103	4.78	−10.73	8.52	6.52	4.78	−3.11	16.14
		MD	1	44	0.09	0.7695	0.17	−1.194	1.20	−3.62	1.23	−0.818	1.20	−3.24	1.61

Cathodal tDCS. Baseline MD of the left FPC significantly interacted with Treatment such that lower MD predicted improvements for tDCS but higher MD predicted sham improvements.

tDCS Group (Cathode)															
DV	Tract	DTI	Interaction (Treatment * Tract)					Active				Sham			
			df1	df2	F	p	R^2	b	SE	CI.low	CI.high	b	SE	CI.low	CI.high
PNT (Untrained)	Left FPC	FA	1	15	2.87	0.1111	###	8.52	5.34	–2.87	19.90	1.97	5.39	–9.52	13.50
		MD	1	15	49.62	<.0001	###	–9.21	1.91	–13.28	–5.13	10.01	1.95	5.86	14.16
	Right FPC	FA	1	44	4.02	0.0511	###	7.47	3.04	1.35	13.60	–0.94	3.17	–7.33	5.45
		MD	1	44	0.51	0.4807	###	–4.26	2.29	–8.88	0.36	–2.12	2.39	–6.94	2.71
	Left PPC	FA	1	31	3.87	0.0581	0.17	4.21	2.27	–0.41	8.84	2.11	2.29	–2.56	6.78
		MD	1	31	5.53	0.0252	0.24	–7.76	2.89	–13.65	–1.86	2.18	3.28	–4.52	8.88
	Right PPC	FA	1	44	2.83	0.0995	0.14	8.50	3.77	0.89	16.10	1.39	4.18	–7.03	9.82
		MD	1	44	2.52	0.1194	0.11	–3.18	2.20	–7.62	1.26	–2.80	2.25	–7.33	1.73
	Left OPC	FA	1	38	1.78	0.1901	0.20	1.06	2.71	–4.43	6.54	6.74	3.34	–0.02	13.50
		MD	1	38	0.15	0.6998	0.13	–0.78	1.90	–4.64	3.08	0.82	3.68	–6.62	8.26
	Right OPC	FA	1	44	0.64	0.4279	0.11	9.92	5.39	–0.94	20.80	4.21	6.24	–8.37	16.80
		MD	1	44	0.04	0.8351	0.14	–3.14	1.99	–7.15	0.87	–3.75	2.15	–8.08	0.59
	Right DRTC	FA	1	43	1.07	0.3076	0.07	1.28	1.50	–1.74	4.30	–0.78	1.54	–3.88	2.31
		MD	1	43	0.54	0.4653	0.06	–0.28	0.50	–1.29	0.74	0.26	0.56	–0.86	1.38
	Left DRTC	FA	1	44	0.72	0.4004	0.08	3.40	3.02	–2.68	9.48	–0.47	3.41	–7.35	6.41
		MD	1	44	0.07	0.8000	0.07	–0.81	0.97	–2.76	1.15	–0.40	1.25	–2.92	2.11

Naming 80 (PNT Trained)

Anodal tDCS. Right PPC significantly interacted with Treatment because lower FA significantly predicted improvements after sham but higher FA trend predicted improvements after tDCS. Baseline MD of the right DRTC significantly interacted with Treatment such that higher MD predicted higher improvements for sham but not tDCS. Overall, these baseline tracts reliably predicted sham improvements (especially the right PPC and right DRTC) but not tDCS improvements (except for right PPC).

tDCS Group (Anode)															
DV	Tract	DTI	nteraction (Treatment * Tract					Active				Sham			
			df1	df2	F	p	R^2	b	SE	CI.low	CI.high	b	SE	CI.low	CI.high
Naming 80 (PNT Trained)	Left FPC	FA	1	26	0.25	0.6183	###	−1.33	2.83	−7.15	4.50	−3.24	2.83	−9.06	2.58
		MD	1	26	0.79	0.3825	###	4.28	3.42	−2.75	11.30	2.67	3.42	−4.36	9.70
	Right FPC	FA	1	44	5.20	0.0275	###	2.27	2.55	−2.87	7.41	−5.79	2.55	−10.93	−0.65
		MD	1	44	6.96	0.0115	###	−3.19	2.38	−7.98	1.60	5.58	2.38	0.79	10.40
	Left PPC	FA	1	38	3.11	0.0858	0.57	2.27	2.88	−3.56	8.09	−4.39	2.88	−10.21	1.44
		MD	1	38	6.40	0.0157	0.59	−2.57	2.37	−7.36	2.23	3.19	2.37	−1.60	7.99
	Right PPC	FA	1	44	7.67	0.0082	0.58	4.91	3.41	−1.96	11.80	−8.27	3.41	−15.14	−1.40
		MD	1	44	3.32	0.0755	0.54	−5.21	3.66	−12.58	2.16	3.70	3.66	−3.67	11.08
	Left OPC	FA	1	32	4.46	0.0426	0.57	2.78	2.33	−1.97	7.53	−3.81	2.33	−8.56	0.94
		MD	1	32	5.06	0.0315	0.58	−1.48	1.18	−3.87	0.92	1.23	1.18	−1.17	3.63
	Right OPC	FA	1	44	5.76	0.0207	0.56	6.36	4.13	−1.97	14.69	−7.07	4.13	−15.40	1.26
		MD	1	44	0.63	0.4326	0.52	−1.57	1.79	−5.17	2.04	0.21	1.79	−3.40	3.81
	Right DRTC	FA	1	38	2.81	0.1019	0.57	1.52	2.59	−3.72	6.75	−4.31	2.59	−9.55	0.92
		MD	1	38	14.09	0.0006	0.65	0.48	3.53	−6.68	7.63	6.74	3.53	−0.42	13.89
	Left DRTC	FA	1	44	4.06	0.0500	0.57	0.41	3.21	−6.06	6.87	−7.87	3.21	−14.34	−1.41
		MD	1	44	0.23	0.6353	0.52	0.11	0.84	−1.59	1.81	0.54	0.84	−1.16	2.24

Cathodal tDCS. Baseline MD of the left FPC significantly interacted with Treatment such that lower MD predicted improvements for tDCS but not sham. Baseline MD of the left DRTC also interacted such that lower MD predicted sham improvements but not for tDCS.

tDCS Group (Cathode)															
DV	Tract	DTI	Interaction (Treatment * Tract)					Active				Sham			
			df1	df2	F	p	R^2	b	SE	CI.low	CI.high	b	SE	CI.low	CI.high
Naming 80 (PNT Trained)	Left FPC	FA	1	16	0.16	0.6924	###	###	7.33	5.40	36.50	###	7.33	3.13	34.20
		MD	1	16	8.28	0.0110	###	−16.0	4.20	−24.89	−7.08	1.10	4.20	−7.81	10.00
	Right FPC	FA	1	45	4.09	0.0490	###	13.50	4.29	4.85	22.10	1.50	4.48	−7.51	10.50
		MD	1	45	0.45	0.5055	###	−8.1	2.97	−14.1	−2.12	−10.7	2.96	−16.6	−4.73
	Left PPC	FA	1	32	1.09	0.3051	0.21	5.27	3.41	−1.67	12.21	0.47	3.44	−6.54	7.48
		MD	1	32	10.41	0.0029	0.37	−13.6	4.06	−21.9	−5.32	−6.4	4.46	−15.5	2.68
	Right PPC	FA	1	45	6.23	0.0163	0.27	18.35	5.16	7.97	28.70	1.55	5.72	−9.96	13.10
		MD	1	45	14.52	0.0004	0.29	−6.91	2.92	−12.8	−1.03	−9.30	2.93	−15.2	−3.39
	Left OPC	FA	1	39	2.50	0.1218	0.15	−2.36	4.28	−11.02	6.29	8.26	5.24	−2.34	18.85
		MD	1	39	1.87	0.1789	0.13	1.43	2.91	−4.46	7.33	−6.72	5.19	−17.22	3.78
	Right OPC	FA	1	45	3.39	0.0723	0.23	22.45	7.46	7.43	37.50	4.36	8.62	−13.01	21.70
		MD	1	45	3.10	0.0853	0.30	−3.42	2.67	−8.80	1.96	−10.00	2.68	−15.40	−4.61
	Right DRTC	FA	1	44	0.05	0.8301	0.11	1.72	2.17	−2.66	6.09	−1.00	2.23	−5.49	3.49
		MD	1	44	3.31	0.0759	0.17	−0.262	0.70	−1.675	1.15	1.59	0.78	0.03	3.15
	Left DRTC	FA	1	45	1.98	0.1667	0.15	−0.345	4.30	−9.004	8.31	8.58	4.68	−0.839	17.99
		MD	1	45	7.50	0.0088	0.22	1.27	1.32	−1.380	3.93	−4.390	1.59	−7.590	−1.190

ASHAFACS (CI)

Anodal tDCS. Baseline MD of the left FPC trend interacted with Treatment such that lower MD predicted higher improvements for tDCS but not sham. Baseline MD of the left OPC significantly interacted with Treatment such that higher MD predicted higher improvements for sham but not for tDCS.

tDCS Group (Anode)															
DV	Tract	DTI	nteraction (Treatment * Tract					Active				Sham			
			df1	df2	F	p	R^2	b	SE	CI.low	CI.high	b	SE	CI.low	CI.high
ASHAFACS (CI)	Left FPC	FA	1	24	2.34	0.1392	###	4.03	2.64	–1.41	9.48	–1.09	2.30	–5.83	3.65
		MD	1	24	7.61	0.0109	###	–4.12	2.79	–9.88	1.64	–0.223	2.72	–5.83	5.39
	Right FPC	FA	1	42	1.43	0.2383	###	1.65	1.83	–2.05	5.34	–1.23	1.60	–4.45	1.99
		MD	1	42	0.10	0.7535	###	–0.545	1.54	–3.66	2.57	0.13	1.52	–2.94	3.20
	Left PPC	FA	1	36	3.03	0.0903	0.39	1.00	1.85	–2.76	4.75	–3.078	1.75	–6.63	0.47
		MD	1	36	0.94	0.3398	0.36	0.08	1.59	–3.13	3.30	1.52	1.55	–1.64	4.67
	Right PPC	FA	1	42	5.32	0.0261	0.41	3.48	2.36	–1.29	8.25	–3.72	2.09	–7.94	0.50
		MD	1	42	0.96	0.3334	0.36	–1.78	2.28	–6.38	2.82	1.19	2.25	–3.34	5.72
	Left OPC	FA	1	31	7.24	0.0114	0.49	0.96	1.43	–1.95	3.88	–4.062	1.38	–6.88	–1.24
		MD	1	31	8.15	0.0076	0.49	–0.142	0.70	–1.56	1.28	1.90	0.70	0.47	3.34
	Right OPC	FA	1	42	0.48	0.4930	0.36	–0.0329	2.72	–5.52	5.45	2.43	2.60	–2.83	7.68
		MD	1	42	0.26	0.6101	0.36	–0.369	1.09	–2.57	1.83	–1.060	1.08	–3.24	1.12
	Right DRTC	FA	1	36	4.24	0.0467	0.41	1.15	1.63	–2.150	4.45	–3.180	1.55	–6.320	–0.039
		MD	1	36	0.41	0.5255	0.36	1.91	2.49	–3.150	6.96	2.65	2.51	–2.430	7.74
	Left DRTC	FA	1	42	1.50	0.2268	0.37	0.76	2.10	–3.480	5.01	–2.511	2.00	–6.550	1.53
		MD	1	42	0.00	0.9847	0.35	0.06	0.51	–0.970	1.10	0.07	0.51	–0.960	1.11

Cathodal tDCS. Baseline FA of the left FPC significantly interacted with Treatment such that higher FA predicted higher improvements for tDCS but not for sham. Baseline MD of the left OPC significantly interacted with Treatment such that lower MD predicted higher improvements for sham but not for tDCS. Baseline MD of the right DRTC significantly interacted with Treatment such that higher MD predicted higher improvements for tDCS but lower MD predicted higher improvements for sham.

tDCS Group (Cathode)															
DV	Tract	DTI	Interaction (Treatment * Tract)					Active				Sham			
			df1	df2	F	p	R^2	b	SE	CI.low	CI.high	b	SE	CI.low	CI.high
ASHAFACS (CI)	Left FPC	FA	1	16	7.71	0.0135	###	8.71	2.76	2.85	14.56	2.83	2.76	-3.02	8.68
		MD	1	16	0.42	0.5256	###	-3.91	1.92	-7.99	0.16	-2.15	1.92	-6.23	1.93
	Right FPC	FA	1	44	1.17	0.2851	###	0.92	2.20	-3.51	5.34	-2.33	2.26	-6.88	2.23
		MD	1	44	1.77	0.1909	###	-2.43	1.57	-5.60	0.74	0.29	1.57	-2.87	3.45
	Left PPC	FA	1	31	4.86	0.0351	0.23	2.08	1.45	-0.89	5.04	-2.14	1.42	-5.03	0.75
		MD	1	31	0.08	0.7744	0.15	-0.68	1.97	-4.69	3.34	-1.50	2.15	-5.88	2.89
	Right PPC	FA	1	44	1.90	0.1746	0.06	0.83	2.67	-4.54	6.21	-4.40	2.92	-10.28	1.48
		MD	1	44	5.61	0.0224	0.12	-1.92	1.47	-4.88	1.04	2.88	1.48	-0.10	5.85
	Left OPC	FA	1	38	0.72	0.4022	0.04	1.22	2.11	-3.05	5.50	-1.54	2.53	-6.66	3.58
		MD	1	38	8.96	0.0048	0.25	-0.46	1.25	-2.99	2.06	-8.04	2.20	-12.49	-3.58
	Right OPC	FA	1	44	1.94	0.1710	0.09	-1.96	3.65	-9.31	5.40	-8.64	4.22	-17.15	-0.13
		MD	1	44	4.38	0.0423	0.11	-2.96	1.36	-5.70	-0.23	1.00	1.36	-1.73	3.74
	Right DRTC	FA	1	43	6.16	0.0171	0.12	-1.59	0.98	-3.57	0.39	1.63	1.00	-0.39	3.64
		MD	1	43	13.28	0.0007	0.22	0.72	0.31	0.09	1.35	-0.91	0.34	-1.59	-0.23
	Left DRTC	FA	1	44	0.16	0.6922	0.07	1.99	2.04	-2.13	6.10	3.18	2.20	-1.25	7.61
		MD	1	44	1.96	0.1683	0.12	-1.54	0.63	-2.81	-0.26	-0.15	0.76	-1.68	1.39

ASHAFACS (QDC)

Anodal tDCS. Baseline FA of the left FPC significantly interacted with Treatment such that higher FA predicted higher improvements for tDCS but not sham. Baseline MD of the right OPC had a significant main effect in general such that higher MD predicted higher improvements across all timepoints and both treatment conditions.

tDCS Group (Anode)																
DV	Tract	DTI	nteraction (Treatment * Tract					Active				Sham				
			df1	df2	F	p	R^2	b	SE	CI.low	CI.high	b	SE	CI.low	CI.high
ASHAFACS (QDC)	Left FPC	FA	1	24	15.79	0.0006	###	11.62	2.40	6.66	16.58	–0.498	2.09	–4.82	3.82
		MD	1	24	1.56	0.2245	###	–8.431	3.23	–15.10	–1.77	–6.390	3.15	–12.90	0.10
	Right FPC	FA	1	42	2.73	0.1061	###	1.95	2.45	–2.99	6.90	–3.353	2.14	–7.66	0.96
		MD	1	42	0.77	0.3855	###	4.76	1.97	0.79	8.73	2.36	1.94	–1.56	6.28
	Left PPC	FA	1	36	0.14	0.7125	0.38	–0.883	2.43	–5.82	4.05	–2.025	2.30	–6.68	2.63
		MD	1	36	0.15	0.6992	0.41	2.38	1.99	–1.66	6.41	3.10	1.95	–0.86	7.06
	Right PPC	FA	1	42	1.62	0.2107	0.33	0.70	3.29	–5.94	7.34	–4.821	2.91	–10.69	1.05
		MD	1	42	2.32	0.1352	0.35	6.02	3.01	–0.05	12.10	–0.093	2.97	–6.08	5.90
	Left OPC	FA	1	31	3.56	0.0687	0.54	–5.660	1.68	–9.09	–2.22	–1.510	1.63	–4.83	1.81
		MD	1	31	1.31	0.2607	0.67	3.71	0.71	2.27	5.15	2.88	0.71	1.42	4.33
	Right OPC	FA	1	42	2.09	0.1556	0.35	–0.668	3.57	–7.87	6.53	–7.417	3.42	–14.31	–0.52
		MD	1	42	0.39	0.5373	0.30	–0.338	1.49	–3.35	2.67	0.81	1.48	–2.17	3.78
	Right DRTC	FA	1	36	0.53	0.4717	0.39	0.24	2.16	–4.13	4.62	2.27	2.05	–1.90	6.44
		MD	1	36	0.38	0.5440	0.40	4.25	3.15	–2.15	10.64	3.35	3.17	–3.08	9.78
	Left DRTC	FA	1	42	2.38	0.1303	0.37	–6.950	2.75	–12.49	–1.41	–1.570	2.61	–6.85	3.70
		MD	1	42	0.46	0.4994	0.40	1.87	0.64	0.58	3.16	1.40	0.64	0.11	2.70

Cathodal tDCS. Baseline MD of the left FPC significantly interacted with Treatment such that lower MD predicted higher improvements for tDCS but not sham.

tDCS Group (Cathode)																
DV	Tract	DTI	Interaction (Treatment * Tract)					Active				Sham				
			df1	df2	F	p	R^2	b	SE	CI.low	CI.high	b	SE	CI.low	CI.high
ASHAFACS (QDC)	Left FPC	FA	1	16	3.74	0.0710	###	6.50	2.76	0.65	12.34	2.41	2.76	-3.44	8.26
		MD	1	16	167.93	<.0001	###	-9.20	0.51	-10.27	-8.13	0.05	0.51	-1.02	1.12
	Right FPC	FA	1	44	3.17	0.0818	###	3.70	1.57	0.53	6.87	-0.12	1.62	-3.38	3.14
		MD	1	44	1.30	0.2599	###	-0.81	1.19	-3.19	1.58	0.95	1.18	-1.43	3.33
	Left PPC	FA	1	31	0.46	0.5050	0.31	0.85	1.27	-1.73	3.43	-0.28	1.23	-2.79	2.24
		MD	1	31	5.11	0.0309	0.48	-5.18	1.42	-8.06	-2.29	-0.58	1.55	-3.73	2.57
	Right PPC	FA	1	44	1.38	0.2464	0.31	3.11	1.99	-0.89	7.11	-0.21	2.17	-4.59	4.16
		MD	1	44	0.00	0.9970	0.30	1.14	1.13	-1.15	3.42	1.14	1.14	-1.15	3.44
	Left OPC	FA	1	38	0.01	0.9192	0.28	-0.15	1.54	-3.26	2.96	0.09	1.84	-3.63	3.82
		MD	1	38	0.03	0.8540	0.43	-2.83	0.91	-4.68	-0.99	-2.49	1.61	-5.75	0.77
	Right OPC	FA	1	44	2.48	0.1223	0.31	3.30	2.75	-2.25	8.85	-2.41	3.19	-8.83	4.02
		MD	1	44	0.45	0.5062	0.29	-0.12	1.05	-2.24	2.01	0.87	1.05	-1.25	3.00
	Right DRTC	FA	1	43	0.08	0.7795	0.36	1.17	0.73	-0.30	2.63	0.90	0.74	-0.59	2.39
		MD	1	43	0.01	0.9203	0.40	-0.49	0.24	-0.97	0.00	-0.45	0.26	-0.97	0.07
	Left DRTC	FA	1	44	0.01	0.9428	0.28	0.74	1.55	-2.39	3.88	0.91	1.68	-2.47	4.28
		MD	1	44	0.28	0.5984	0.28	0.00	0.49	-0.99	1.00	0.41	0.60	-0.79	1.61

Appendix 4A

Baseline tracts (bilateral MCP and SCP) predicting language outcomes for both tDCS groups combined

DV	Tract	DTI	Interaction (Treatment * Tract)					Active				Sham			
			df1	df2	F	p	R^2	b	SE	CI.low	CI.high	b	SE	CI.low	CI.high
PNT (Untrained)	Left MCP	FA	1	96	0.50	0.4829	0.07	0.58	2.76	-4.89	6.05	3.37	3.04	-2.65	9.40
		MD	1	96	0.25	0.6182	0.09	-1.93	1.70	-5.28	1.42	-3.02	1.69	-6.37	0.33
	Right MCP	FA	1	96	0.83	0.3656	0.08	0.97	2.42	-3.83	5.76	3.95	2.46	-0.94	8.84
		MD	1	96	4.52	0.0360	0.13	-4.57	1.57	-7.68	-1.46	0.01	1.61	-3.18	3.20
	Left SCP	FA	1	96	1.30	0.2566	0.08	0.04	1.71	-3.35	3.44	-2.70	1.89	-6.45	1.04
		MD	1	96	0.56	0.4558	0.07	-0.02	0.31	-0.63	0.59	0.30	0.32	-0.34	0.95
	Right SCP	FA	1	96	0.00	0.9719	0.06	-0.02	1.29	-2.57	2.53	0.04	1.30	-2.54	2.62
		MD	1	96	0.01	0.9327	0.06	-0.03	0.45	-0.92	0.86	0.02	0.46	-0.89	0.93

DV	Tract	DTI	Interaction (Treatment * Tract)					Active				Sham			
			df1	df2	F	p	R^2	b	SE	CI.low	CI.high	b	SE	CI.low	CI.high
Naming 80 (PNT Trained)	Left MCP	FA	1	97	7.20	0.0086	0.26	8.85	3.10	2.69	15.01	-3.11	3.41	-9.88	3.66
		MD	1	97	0.16	0.6928	0.23	-3.68	1.96	-7.58	0.22	-2.68	1.96	-6.57	1.22
	Right MCP	FA	1	97	0.01	0.9081	0.20	-0.15	2.85	-5.81	5.51	0.30	2.88	-5.41	6.01
		MD	1	97	4.41	0.0384	0.25	0.17	1.84	-3.48	3.81	-5.08	1.84	-8.73	-1.43
	Left SCP	FA	1	97	2.40	0.1243	0.22	-0.10	1.98	-4.03	3.82	4.19	2.17	-0.11	8.49
		MD	1	97	0.61	0.4368	0.20	0.01	0.36	-0.69	0.72	-0.38	0.38	-1.13	0.37
	Right SCP	FA	1	97	1.22	0.2718	0.22	-0.20	1.48	-3.14	2.75	-2.38	1.50	-5.36	0.60
		MD	1	97	2.60	0.1102	0.25	0.25	0.51	-0.75	1.25	1.37	0.51	0.35	2.39

DV	Tract	DTI	Interaction (Treatment * Tract)					Active				Sham			
			df1	df2	F	p	R^2	b	SE	CI.low	CI.high	b	SE	CI.low	CI.high
ASHAFACS (CI)	Left MCP	FA	1	94	2.59	0.1108	0.07	2.03	1.58	-1.11	5.17	-1.57	1.69	-4.92	1.79
		MD	1	94	14.72	0.0002	0.19	-3.59	0.89	-5.36	-1.81	0.81	0.89	-0.96	2.57
	Right MCP	FA	1	94	0.86	0.3563	0.07	2.09	1.41	-0.72	4.90	0.36	1.37	-2.37	3.08
		MD	1	94	0.10	0.7530	0.07	-1.16	0.91	-2.97	0.65	-0.77	0.91	-2.57	1.03
	Left SCP	FA	1	94	0.22	0.6387	0.11	2.20	0.96	0.29	4.11	1.57	1.03	-0.47	3.61
		MD	1	94	0.99	0.3227	0.11	-0.44	0.17	-0.77	-0.10	-0.20	0.18	-0.55	0.15
	Right SCP	FA	1	94	2.63	0.1081	0.07	-0.67	0.73	-2.12	0.79	0.88	0.72	-0.55	2.32
		MD	1	94	5.39	0.0224	0.09	0.41	0.25	-0.09	0.91	-0.38	0.25	-0.87	0.11

DV	Tract	DTI	Interaction (Treatment * Tract)					Active				Sham			
			df1	df2	F	p	R^2	b	SE	CI.low	CI.high	b	SE	CI.low	CI.high
ASHAFACS (QDC)	Left MCP	FA	1	94	0.94	0.3349	0.24	0.38	1.55	-2.70	3.47	-1.75	1.66	-5.04	1.55
		MD	1	94	1.25	0.2668	0.25	0.74	0.94	-1.13	2.60	-0.61	0.94	-2.47	1.25
	Right MCP	FA	1	94	0.03	0.8682	0.24	0.29	1.39	-2.48	3.06	-0.02	1.35	-2.70	2.66
		MD	1	94	0.48	0.4916	0.31	-1.70	0.85	-3.39	-0.01	-2.50	0.85	-4.18	-0.82
	Left SCP	FA	1	94	0.07	0.7960	0.25	-0.71	0.96	-2.63	1.20	-1.06	1.03	-3.10	0.99
		MD	1	94	0.00	0.9593	0.24	0.13	0.17	-0.21	0.46	0.14	0.18	-0.21	0.49
	Right SCP	FA	1	94	0.68	0.4114	0.25	-1.05	0.72	-2.47	0.38	-0.28	0.71	-1.68	1.13
		MD	1	94	2.31	0.1320	0.27	0.52	0.25	0.03	1.02	0.02	0.24	-0.47	0.50

Appendix 4B

Baseline tracts (bilateral MCP and SCP) predicting language outcomes for separate tDCS groups

PNT (Untrained)

tDCS Group (Anode)															
DV	Tract	DTI	nteraction (Treatment * Tract					Active				Sham			
			df1	df2	F	p	R^2	b	SE	CI.low	CI.high	b	SE	CI.low	CI.high
PNT (Untrained)	Left MCP	FA	1	44	5.03	0.0301	###	###	4.69	-15.12	3.77	8.54	4.69	-0.90	17.98
		MD	1	44	0.03	0.8592	###	-3.10	3.40	-9.96	3.76	-3.86	3.40	-10.72	3.00
	Right MCP	FA	1	44	3.39	0.0722	###	-8.43	7.17	-22.89	6.02	7.46	7.17	-6.99	21.92
		MD	1	44	6.49	0.0144	###	-4.89	2.25	-9.44	-0.35	2.22	2.25	-2.33	6.76
	Left SCP	FA	1	44	0.00	0.9769	0.16	2.71	3.64	-4.62	10.00	2.82	3.64	-4.51	10.20
		MD	1	44	0.13	0.7256	0.16	-0.45	0.50	-1.46	0.57	-0.25	0.50	-1.26	0.76
	Right SCP	FA	1	44	1.11	0.2971	0.17	-2.59	2.93	-8.49	3.31	1.43	2.93	-4.48	7.33
		MD	1	44	0.00	0.9621	0.16	-0.51	0.95	-2.41	1.40	-0.45	0.95	-2.35	1.46

tDCS Group (Cathode)															
DV	Tract	DTI	nteraction (Treatment * Tract					Active				Sham			
			df1	df2	F	p	R^2	b	SE	CI.low	CI.high	b	SE	CI.low	CI.high
PNT (Untrained)	Left MCP	FA	1	44	1.09	0.3018	###	5.45	3.04	-0.69	11.58	0.81	3.54	-6.32	7.94
		MD	1	44	0.29	0.5950	###	-2.35	1.67	-5.71	1.01	-3.53	1.71	-6.98	-0.08
	Right MCP	FA	1	44	0.02	0.8855	###	2.52	2.26	-2.03	7.07	2.97	2.32	-1.70	7.65
		MD	1	44	0.01	0.9288	###	-2.10	2.38	-6.90	2.70	-1.81	2.56	-6.98	3.36
	Left SCP	FA	1	44	2.93	0.0938	0.10	1.84	1.85	-1.89	5.57	-3.13	2.37	-7.90	1.65
		MD	1	44	0.94	0.3382	0.07	-0.38	0.47	-1.32	0.56	0.34	0.59	-0.84	1.52
	Right SCP	FA	1	44	0.67	0.4184	0.07	1.10	1.34	-1.61	3.80	-0.36	1.39	-3.17	2.45
		MD	1	44	0.12	0.7289	0.06	-0.08	0.51	-1.11	0.95	0.17	0.55	-0.94	1.28

Naming 80 (PNT Trained)

tDCS Group (Anode)																
DV	Tract	DTI	nteraction (Treatment * Tract					Active				Sham				
			df1	df2	F	p	R^2	b	SE	CI.low	CI.high	b	SE	CI.low	CI.high
Naming 80 (PNT Trained)	Left MCP	FA	1	44	6.80	0.0124	###	3.84	3.21	-2.62	10.30	###	3.21	-13.93	-1.01
		MD	1	44	0.95	0.3360	###	0.31	2.37	-4.47	5.08	3.21	2.37	-1.56	7.99
	Right MCP	FA	1	44	0.82	0.3689	###	1.61	5.11	-8.69	11.90	-3.97	5.11	-14.27	6.32
		MD	1	44	3.36	0.0734	###	1.54	1.62	-1.72	4.80	-2.14	1.62	-5.40	1.13
	Left SCP	FA	1	44	0.02	0.8787	0.52	-1.07	2.55	-6.20	4.07	-1.47	2.55	-6.60	3.66
		MD	1	44	0.14	0.7130	0.52	0.06	0.35	-0.65	0.77	-0.09	0.35	-0.79	0.62
	Right SCP	FA	1	44	0.13	0.7156	0.52	-1.78	2.05	-5.90	2.35	-0.80	2.05	-4.93	3.32
		MD	1	44	0.13	0.7194	0.52	0.31	0.66	-1.02	1.64	0.00	0.66	-1.33	1.33

tDCS Group (Cathode)																
DV	Tract	DTI	nteraction (Treatment * Tract					Active				Sham				
			df1	df2	F	p	R^2	b	SE	CI.low	CI.high	b	SE	CI.low	CI.high
Naming 80 (PNT Trained)	Left MCP	FA	1	45	4.61	0.0372	###	9.53	4.32	0.83	18.23	###	5.01	-14.04	6.14
		MD	1	45	0.13	0.7182	###	-4.69	2.36	-9.44	0.07	-5.82	2.41	-10.68	-0.96
	Right MCP	FA	1	45	0.65	0.4230	###	-0.59	3.33	-7.30	6.11	3.12	3.37	-3.67	9.90
		MD	1	45	3.27	0.0774	###	-2.51	3.19	-8.93	3.90	-10.09	3.19	-16.50	-3.67
	Left SCP	FA	1	45	5.17	0.0279	0.24	0.93	2.54	-4.18	6.04	9.79	3.14	3.47	16.11
		MD	1	45	3.96	0.0528	0.19	0.03	0.65	-1.28	1.33	-1.99	0.80	-3.59	-0.39
	Right SCP	FA	1	45	0.07	0.7901	0.10	1.04	1.96	-2.90	4.98	0.34	2.03	-3.75	4.43
		MD	1	45	0.30	0.5851	0.12	0.26	0.73	-1.22	1.74	0.83	0.79	-0.76	2.42

ASHAFACS (CI)

tDCS Group (Anode)																
DV	Tract	DTI	nteraction (Treatment * Tract					Active				Sham				
			df1	df2	F	p	R^2	b	SE	CI.low	CI.high	b	SE	CI.low	CI.high
ASHAFACS (CI)	Left MCP	FA	1	42	5.85	0.0200	###	2.89	2.12	-1.39	7.16	###	1.94	-7.74	0.10
		MD	1	42	4.73	0.0354	###	-1.53	1.41	-4.37	1.32	2.28	1.39	-0.52	5.08
	Right MCP	FA	1	42	9.98	0.0029	###	3.86	3.02	-2.23	9.95	-7.29	2.83	-13.01	-1.57
		MD	1	42	2.96	0.0926	###	-1.41	0.98	-3.39	0.57	0.67	0.98	-1.31	2.65
	Left SCP	FA	1	42	0.02	0.8900	0.35	-1.20	1.57	-4.37	1.97	-0.97	1.54	-4.08	2.13
		MD	1	42	0.01	0.9176	0.35	0.09	0.22	-0.35	0.52	0.11	0.21	-0.32	0.54
	Right SCP	FA	1	42	3.16	0.0829	0.40	0.25	1.25	-2.27	2.78	-2.53	1.19	-4.93	-0.12
		MD	1	42	0.95	0.3359	0.36	-0.07	0.43	-0.93	0.79	0.45	0.39	-0.35	1.24

tDCS Group (Cathode)																
DV	Tract	DTI	nteraction (Treatment * Tract					Active				Sham				
			df1	df2	F	p	R^2	b	SE	CI.low	CI.high	b	SE	CI.low	CI.high
ASHAFACS (CI)	Left MCP	FA	1	44	0.06	0.8037	###	###	2.16	-5.47	3.22	###	2.46	-6.86	3.06
		MD	1	44	12.25	0.0011	###	-1.99	1.07	-4.15	0.17	2.97	1.10	0.76	5.18
	Right MCP	FA	1	44	0.01	0.9155	###	1.38	1.62	-1.89	4.64	1.14	1.58	-2.04	4.32
		MD	1	44	1.44	0.2371	###	-0.01	1.60	-3.23	3.21	-2.53	1.59	-5.74	0.68
	Left SCP	FA	1	44	1.10	0.2996	0.05	1.64	1.30	-0.98	4.26	-0.45	1.58	-3.63	2.73
		MD	1	44	6.81	0.0123	0.14	-0.69	0.31	-1.31	-0.07	0.55	0.37	-0.19	1.29
	Right SCP	FA	1	44	4.70	0.0356	0.10	-1.19	0.89	-2.98	0.60	1.35	0.92	-0.49	3.20
		MD	1	44	7.68	0.0081	0.15	0.71	0.33	0.05	1.37	-0.56	0.35	-1.26	0.14

ASHAFACS (QDC)

tDCS Group (Anode)																
DV	Tract	DTI	nteraction (Treatment * Tract					Active				Sham				
			df1	df2	F	p	R^2	b	SE	CI.low	CI.high	b	SE	CI.low	CI.high
ASHAFACS (QDC)	Left MCP	FA	1	42	0.02	0.8767	###	###	2.83	-11.10	0.35	###	2.59	-11.20	-0.71
		MD	1	42	8.05	0.0070	###	5.28	1.83	1.58	8.98	-1.19	1.81	-4.84	2.45
	Right MCP	FA	1	42	0.00	0.9672	###	-5.39	4.40	-14.30	3.50	-5.60	4.13	-13.90	2.74
		MD	1	42	1.78	0.1895	###	-4.31	1.02	-6.37	-2.26	-5.98	1.02	-8.03	-3.93
	Left SCP	FA	1	42	2.30	0.1368	0.49	-7.87	1.84	-11.57	-4.16	-5.01	1.80	-8.64	-1.38
		MD	1	42	1.36	0.2496	0.48	1.05	0.25	0.54	1.55	0.73	0.25	0.23	1.23
	Right SCP	FA	1	42	6.95	0.0117	0.42	-5.41	1.61	-8.66	-2.16	-0.11	1.53	-3.21	2.98
		MD	1	42	13.23	0.0007	0.49	2.14	0.50	1.13	3.15	-0.13	0.46	-1.07	0.80

tDCS Group (Cathode)																
DV	Tract	DTI	nteraction (Treatment * Tract					Active				Sham				
			df1	df2	F	p	R^2	b	SE	CI.low	CI.high	b	SE	CI.low	CI.high
ASHAFACS (QDC)	Left MCP	FA	1	44	1.22	0.2758	###	2.46	1.58	-0.73	5.64	###	1.80	-3.68	3.59
		MD	1	44	0.11	0.7443	###	1.38	0.84	-0.32	3.07	1.74	0.86	0.01	3.48
	Right MCP	FA	1	44	0.17	0.6799	###	1.00	1.21	-1.45	3.44	0.32	1.18	-2.06	2.70
		MD	1	44	0.21	0.6491	###	-0.59	1.21	-3.04	1.85	0.14	1.21	-2.30	2.58
	Left SCP	FA	1	44	2.13	0.1512	0.31	0.24	0.96	-1.69	2.17	-1.90	1.16	-4.25	0.44
		MD	1	44	2.60	0.1142	0.34	0.02	0.23	-0.44	0.49	0.61	0.28	0.04	1.18
	Right SCP	FA	1	44	0.34	0.5627	0.32	1.10	0.67	-0.26	2.45	0.58	0.69	-0.82	1.97
		MD	1	44	0.05	0.8295	0.31	-0.32	0.26	-0.84	0.19	-0.25	0.27	-0.80	0.31

Appendix 5A

Baseline tracts (bilateral frontal, parietal, occipital corticopontine tracts) predicting language outcomes for combined tDCS groups

PNT (Untrained)

DV	Tract	DTI	Interaction (Treatment * Tract					tDCS				Sham			
			df1	df2	F	p	R^2	b	SE	CI.low	CI.high	b	SE	CI.low	CI.high
PNT (Untrained)	Left frontal CPT	FA	1	49	3.83	0.0560	0.22	0.80	1.85	-2.92	4.53	###	1.85	-7.10	0.33
		MD	1	49	7.95	0.0069	0.28	-0.85	1.12	-3.10	1.41	3.24	1.12	0.99	5.49
	Right frontal CPT	FA	1	96	0.61	0.4354	0.07	0.73	1.81	-2.86	4.33	-1.25	1.83	-4.88	2.38
		MD	1	96	0.04	0.8337	0.06	0.25	1.25	-2.22	2.73	-0.11	1.28	-2.65	2.42
	Left parietal CPT	FA	1	77	0.26	0.6120	0.12	0.52	1.45	-2.37	3.41	1.52	1.49	-1.44	4.48
		MD	1	77	6.82	0.0108	0.17	1.33	0.84	-0.35	3.00	-1.67	0.85	-3.36	0.02
	Right parietal CPT	FA	1	96	0.01	0.9204	0.07	1.55	2.22	-2.85	5.96	1.24	2.24	-3.21	5.69
		MD	1	96	0.37	0.5451	0.07	-1.42	1.38	-4.16	1.32	-0.25	1.39	-3.01	2.51
	Left occipital CPT	FA	1	78	8.86	0.0039	0.19	-2.11	1.44	-4.98	0.76	4.09	1.57	0.96	7.21
		MD	1	78	10.30	0.0019	0.21	0.56	0.54	-0.51	1.63	-1.65	0.55	-2.75	-0.55
	Right occipital CPT	FA	1	96	0.61	0.4362	0.07	-0.04	2.93	-5.87	5.78	-3.22	3.07	-9.31	2.86
		MD	1	96	1.06	0.3060	0.07	-1.15	1.00	-3.13	0.83	0.25	1.04	-1.81	2.31
	Right nd-DRTC	FA	1	89	0.14	0.7057	0.08	-0.23	1.29	-2.79	2.33	0.46	1.34	-2.20	3.12
		MD	1	89	0.42	0.5171	0.08	0.13	0.45	-0.77	1.02	-0.32	0.54	-1.40	0.76
	Left nd-DRTC	FA	1	96	3.00	0.0864	0.10	-0.45	2.50	-5.42	4.52	5.45	2.51	0.47	10.44
		MD	1	96	0.91	0.3414	0.07	0.05	1.72	-3.36	3.46	-2.26	1.78	-5.80	1.28

Naming 80 (PNT Trained)

DV	Tract	DTI	Interaction (Treatment * Tract					tDCS				Sham			
			df1	df2	F	p	R^2	b	SE	CI.low	CI.high	b	SE	CI.low	CI.high
Naming 80 (trained)	Left frontal CPT	FA	1	50	2.81	0.0998	0.18	5.62	2.39	0.83	10.42	0.99	2.39	-3.81	5.79
		MD	1	50	6.76	0.0122	0.22	-4.31	1.46	-7.25	-1.38	0.61	1.46	-2.33	3.55
	Right frontal CPT	FA	1	97	5.74	0.0186	0.24	4.20	2.06	0.11	8.30	-2.72	2.08	-6.85	1.41
		MD	1	97	1.10	0.2963	0.21	-0.07	1.44	-2.93	2.79	2.03	1.45	-0.86	4.91
	Left parietal CPT	FA	1	78	4.66	0.0339	0.26	5.13	1.76	1.62	8.63	0.00	1.79	-3.57	3.57
		MD	1	78	13.46	0.0004	0.30	-2.03	1.03	-4.07	0.01	3.10	1.03	1.04	5.16
	Right parietal CPT	FA	1	97	6.39	0.0131	0.25	6.65	2.51	1.67	11.60	-2.33	2.53	-7.35	2.70
		MD	1	97	0.22	0.6398	0.20	-0.78	1.62	-3.99	2.43	0.28	1.62	-2.94	3.50
	Left occipital CPT	FA	1	79	0.92	0.3411	0.21	0.97	1.88	-2.77	4.72	-1.63	2.04	-5.69	2.44
		MD	1	79	5.82	0.0182	0.25	-0.85	0.69	-2.22	0.53	1.29	0.71	-0.12	2.71
	Right occipital CPT	FA	1	97	1.70	0.1952	0.21	2.26	3.41	-4.52	9.03	-3.90	3.55	-10.94	3.14
		MD	1	97	0.85	0.3603	0.21	0.02	1.16	-2.29	2.33	-1.41	1.17	-3.73	0.91
	Right nd-DRTC	FA	1	90	1.05	0.3085	0.20	2.29	1.55	-0.79	5.37	0.06	1.61	-3.14	3.26
		MD	1	90	6.07	0.0156	0.24	-1.16	0.53	-2.22	-0.11	0.83	0.64	-0.44	2.11
	Left nd-DRTC	FA	1	97	0.75	0.3885	0.20	1.50	2.98	-4.41	7.41	-2.00	2.98	-7.91	3.91
		MD	1	97	0.27	0.6070	0.20	1.38	2.01	-2.61	5.38	-0.05	2.04	-4.10	4.00

ASHAFACS (CI)

DV	Tract	DTI	Interaction (Treatment * Tract)					tDCS				Sham			
			df1	df2	F	p	R^2	b	SE	CI.low	CI.high	b	SE	CI.low	CI.high
ASHAFACS (CI)	Left frontal CPT	FA	1	48	5.48	0.0235	0.26	4.16	1.08	1.98	6.33	1.31	1.05	-0.79	3.42
		MD	1	48	0.61	0.4390	0.15	-1.51	0.74	-3.00	-0.01	-0.78	0.71	-2.20	0.65
	Right frontal CPT	FA	1	94	1.31	0.2546	0.06	1.50	1.08	-0.64	3.64	-0.18	1.02	-2.20	1.85
		MD	1	94	4.10	0.0458	0.09	0.59	0.70	-0.79	1.97	-1.35	0.69	-2.72	0.03
	Left parietal CPT	FA	1	75	9.13	0.0034	0.16	0.64	0.78	-0.92	2.20	-2.53	0.79	-4.10	-0.95
		MD	1	75	0.00	0.9570	0.09	0.72	0.50	-0.27	1.72	0.76	0.49	-0.21	1.73
	Right parietal CPT	FA	1	94	3.48	0.0654	0.08	1.49	1.25	-1.00	3.97	-1.79	1.24	-4.25	0.67
		MD	1	94	1.47	0.2278	0.06	1.02	0.78	-0.54	2.57	-0.29	0.77	-1.83	1.24
	Left occipital CPT	FA	1	77	3.67	0.0590	0.12	0.17	0.91	-1.65	1.98	-2.31	0.96	-4.22	-0.40
		MD	1	77	0.51	0.4775	0.09	0.22	0.34	-0.46	0.90	0.53	0.35	-0.17	1.23
	Right occipital CPT	FA	1	94	0.71	0.4017	0.06	0.37	1.67	-2.95	3.69	2.30	1.71	-1.10	5.69
		MD	1	94	1.43	0.2348	0.14	-0.80	0.54	-1.88	0.27	-1.66	0.54	-2.73	-0.59
	Right nd-DRTC	FA	1	87	0.76	0.3849	0.05	-0.90	0.75	-2.39	0.58	0.01	0.78	-1.53	1.55
		MD	1	87	4.62	0.0343	0.09	0.52	0.26	0.01	1.03	-0.33	0.31	-0.94	0.29
	Left nd-DRTC	FA	1	94	0.10	0.7557	0.05	0.34	1.48	-2.60	3.28	-0.28	1.44	-3.13	2.58
		MD	1	94	2.48	0.1186	0.08	0.43	0.96	-1.48	2.33	-1.66	0.97	-3.58	0.27

ASHAFACS (QDC)

DV	Tract	DTI	Interaction (Treatment * Tract)					tDCS				Sham			
			df1	df2	F	p	R^2	b	SE	CI.low	CI.high	b	SE	CI.low	CI.high
ASHAFACS (QDC)	Left frontal CPT	FA	1	48	22.02	<.0001	0.55	5.12	0.90	3.31	6.93	0.38	0.87	-1.37	2.13
		MD	1	48	36.62	<.0001	0.58	-3.16	0.55	-4.27	-2.05	1.05	0.53	-0.01	2.12
	Right frontal CPT	FA	1	94	7.66	0.0068	0.30	2.95	1.02	0.93	4.96	-0.88	0.96	-2.79	1.03
		MD	1	94	1.73	0.1915	0.28	0.37	0.68	-0.97	1.71	1.59	0.67	0.26	2.92
	Left parietal CPT	FA	1	75	0.51	0.4787	0.25	0.44	0.84	-1.23	2.11	-0.36	0.85	-2.05	1.32
		MD	1	75	2.98	0.0884	0.33	0.39	0.49	-0.58	1.36	1.52	0.47	0.57	2.46
	Right parietal CPT	FA	1	94	2.63	0.1083	0.26	2.00	1.22	-0.43	4.43	-0.78	1.21	-3.19	1.62
		MD	1	94	0.33	0.5658	0.25	0.43	0.76	-1.09	1.94	1.03	0.75	-0.46	2.53
	Left occipital CPT	FA	1	77	3.67	0.0591	0.39	-2.93	0.79	-4.51	-1.35	-0.78	0.83	-2.44	0.87
		MD	1	77	0.25	0.6175	0.40	1.00	0.29	0.43	1.57	0.82	0.29	0.23	1.40
	Right occipital CPT	FA	1	94	7.66	0.0068	0.30	2.95	1.02	0.93	4.96	-0.88	0.96	-2.79	1.03
		MD	1	94	1.73	0.1915	0.28	0.37	0.68	-0.97	1.71	1.59	0.67	0.26	2.92
	Right nd-DRTC	FA	1	87	0.02	0.8785	0.26	-0.41	0.71	-1.82	1.01	-0.56	0.74	-2.03	0.92
		MD	1	87	2.36	0.1282	0.28	-0.02	0.25	-0.52	0.47	0.56	0.30	-0.03	1.15
	Left nd-DRTC	FA	1	94	3.50	0.0646	0.27	-3.14	1.41	-5.94	-0.33	0.39	1.37	-2.33	3.11
		MD	1	94	0.19	0.6672	0.29	2.14	0.92	0.32	3.96	1.59	0.93	-0.24	3.43

Appendix 5B

Baseline tracts (bilateral frontal, parietal, occipital corticopontine tracts) predicting language outcomes for separate tDCS groups

PNT (Untrained)

tDCS Group (Anode)																
DV	Tract	DTI	nteraction (Treatment * Tract					tDCS				Sham				
			df1	df2	F	p	R^2	b	SE	CI.low	CI.high	b	SE	CI.low	CI.high
PNT (Untrained)	Left frontal CPT	FA	1	26	0.14	0.7118	0.34	###	2.45	-5.46	4.61	###	2.45	-6.71	3.37
		MD	1	26	0.32	0.5778	0.36	0.57	1.44	-2.38	3.52	1.68	1.44	-1.27	4.63
	Right frontal CPT	FA	1	44	0.83	0.3664	0.17	-2.25	2.71	-7.70	3.21	1.18	2.71	-4.28	6.63
		MD	1	44	3.14	0.0833	0.20	2.25	2.08	-1.94	6.43	-2.56	2.08	-6.74	1.63
	Left parietal CPT	FA	1	38	5.95	0.0195	0.28	-4.75	2.44	-9.68	0.19	3.01	2.44	-1.92	7.95
		MD	1	38	15.13	0.0004	0.39	2.78	0.97	0.82	4.75	-1.77	0.97	-3.73	0.20
	Right parietal CPT	FA	1	44	2.65	0.1104	0.19	-5.54	4.34	-14.28	3.20	3.83	4.34	-4.91	12.60
		MD	1	44	0.01	0.9282	0.16	-0.85	2.83	-6.55	4.85	-1.17	2.83	-6.87	4.54
	Left occipital CPT	FA	1	32	6.76	0.0140	0.32	-2.50	1.85	-6.28	1.28	3.89	1.85	0.12	7.67
		MD	1	32	12.82	0.0011	0.39	1.14	0.69	-0.25	2.54	-1.62	0.69	-3.01	-0.22
	Right occipital CPT	FA	1	44	0.72	0.4024	0.17	3.34	3.86	-4.45	11.10	-0.79	3.86	-8.57	7.00
		MD	1	44	0.98	0.3288	0.17	-1.49	1.49	-4.48	1.51	0.32	1.49	-2.68	3.31
	Right nd-DRTC	FA	1	38	7.56	0.0091	0.30	-4.97	2.42	-9.86	-0.08	3.82	2.42	-1.07	8.71
		MD	1	38	12.78	0.0010	0.37	3.07	1.09	0.87	5.28	-1.06	1.09	-3.26	1.15
	Left nd-DRTC	FA	1	44	6.59	0.0137	0.25	-4.77	3.91	-12.65	3.12	8.86	3.91	0.98	16.74
		MD	1	44	1.60	0.2133	0.19	0.32	2.98	-5.67	6.32	-4.65	2.98	-10.64	1.35

tDCS Group (Cathode)																
DV	Tract	DTI	Interaction (Treatment * Tract					tDCS				Sham				
			df1	df2	F	p	R^2	b	SE	CI.low	CI.high	b	SE	CI.low	CI.high
PNT (Untrained)	Left frontal CPT	FA	1	15	6.09	0.0261	0.33	2.72	3.94	-5.68	11.12	###	3.92	-11.54	5.15
		MD	1	15	21.10	0.0004	0.57	-2.81	1.10	-5.16	-0.45	4.20	1.11	1.83	6.57
	Right frontal CPT	FA	1	44	6.92	0.0117	0.18	5.40	2.14	1.09	9.71	-2.45	2.17	-6.82	1.92
		MD	1	44	2.84	0.0991	0.10	-1.87	1.42	-4.73	1.00	1.59	1.51	-1.46	4.65
	Left parietal CPT	FA	1	31	1.57	0.2192	0.15	2.71	1.61	-0.58	6.00	-0.09	1.66	-3.47	3.30
		MD	1	31	5.35	0.0275	0.22	-4.06	1.75	-7.64	-0.49	2.00	1.94	-1.96	5.96
	Right parietal CPT	FA	1	44	2.53	0.1187	0.11	4.20	2.32	-0.46	8.87	-1.03	2.36	-5.78	3.73
		MD	1	44	1.06	0.3080	0.07	-1.30	1.48	-4.29	1.70	0.88	1.51	-2.16	3.92
	Left occipital CPT	FA	1	38	1.90	0.1758	0.16	-1.38	2.16	-5.76	2.99	4.69	3.82	-3.04	12.43
		MD	1	38	1.14	0.2928	0.15	0.04	0.94	-1.86	1.94	3.42	3.03	-2.72	9.56
	Right occipital CPT	FA	1	44	0.01	0.9266	0.05	1.24	6.41	-11.70	14.10	0.49	7.11	-13.80	14.80
		MD	1	44	0.18	0.6771	0.08	-1.44	1.32	-4.09	1.22	-0.58	1.55	-3.71	2.54
	Right nd-DRTC	FA	1	43	1.33	0.2554	0.07	1.15	1.57	-2.02	4.32	-1.29	1.61	-4.54	1.95
		MD	1	43	1.77	0.1901	0.08	-0.46	0.50	-1.46	0.54	0.57	0.61	-0.66	1.79
	Left nd-DRTC	FA	1	44	0.01	0.9115	0.06	1.01	2.83	-4.70	6.72	1.46	2.85	-4.29	7.21
		MD	1	44	0.02	0.8933	0.06	-0.30	1.89	-4.11	3.52	-0.67	2.06	-4.82	3.48

Naming 80 (PNT Trained)

tDCS Group (Anode)																
DV	Tract	DTI	nteraction (Treatment * Tract					tDCS				Sham				
			df1	df2	F	p	R^2	b	SE	CI.low	CI.high	b	SE	CI.low	CI.high
Naming 80 (trained)	Left frontal CPT	FA	1	26	1.50	0.2315	0.67	0.58	1.59	-2.68	3.84	###	1.59	-5.32	1.20
		MD	1	26	0.06	0.8159	0.65	-0.29	0.96	-2.26	1.69	-0.59	0.96	-2.57	1.38
	Right frontal CPT	FA	1	44	2.93	0.0939	0.55	0.93	1.84	-2.77	4.63	-3.43	1.84	-7.14	0.27
		MD	1	44	5.90	0.0193	0.56	-1.95	1.41	-4.80	0.90	2.53	1.41	-0.32	5.37
	Left parietal CPT	FA	1	38	4.59	0.0386	0.58	1.78	1.80	-1.86	5.42	-3.25	1.80	-6.89	0.39
		MD	1	38	9.62	0.0036	0.61	-1.52	0.74	-3.03	-0.02	1.25	0.74	-0.25	2.75
	Right parietal CPT	FA	1	44	5.25	0.0268	0.56	3.34	2.95	-2.60	9.29	-5.62	2.95	-11.60	0.32
		MD	1	44	1.01	0.3204	0.54	-3.30	1.92	-7.18	0.58	-0.93	1.92	-4.80	2.95
	Left occipital CPT	FA	1	32	5.49	0.0255	0.58	1.73	1.35	-1.02	4.47	-2.46	1.35	-5.20	0.29
		MD	1	32	8.18	0.0074	0.60	-0.93	0.51	-1.98	0.12	0.72	0.51	-0.32	1.77
	Right occipital CPT	FA	1	44	0.57	0.4551	0.52	2.20	2.69	-3.23	7.63	-0.36	2.69	-5.79	5.07
		MD	1	44	0.40	0.5327	0.53	-1.15	1.04	-3.24	0.94	-0.35	1.04	-2.44	1.74
	Right nd-DRTC	FA	1	38	6.21	0.0171	0.59	2.17	1.78	-1.43	5.77	-3.70	1.78	-7.30	-0.10
		MD	1	38	9.74	0.0034	0.61	-1.65	0.82	-3.32	0.01	1.06	0.82	-0.60	2.73
	Left nd-DRTC	FA	1	44	9.32	0.0038	0.59	2.95	2.64	-2.37	8.27	-7.99	2.64	-13.32	-2.67
		MD	1	44	5.90	0.0193	0.57	-4.32	2.00	-8.36	-0.28	2.12	2.00	-1.92	6.16

tDCS Group (Cathode)															
DV	Tract	DTI	Interaction (Treatment * Tract					tDCS				Sham			
			df1	df2	F	p	R^2	b	SE	CI.low	CI.high	b	SE	CI.low	CI.high
Naming 80 (trained)	Left frontal CPT	FA	1	16	3.77	0.0699	0.42	###	5.82	4.22	28.90	9.61	5.82	-2.72	21.90
		MD	1	16	18.36	0.0006	0.55	-6.47	1.80	-10.29	-2.66	4.12	1.80	0.31	7.93
	Right frontal CPT	FA	1	45	2.87	0.0972	0.25	9.75	3.04	3.63	15.87	2.56	3.08	-3.63	8.76
		MD	1	45	0.41	0.5261	0.21	-2.84	1.99	-6.84	1.16	-4.63	2.00	-8.66	-0.60
	Left parietal CPT	FA	1	32	6.15	0.0186	0.29	5.37	2.27	0.74	9.99	-2.30	2.29	-6.96	2.36
		MD	1	32	16.42	0.0003	0.44	-9.17	2.29	-13.83	-4.51	4.67	2.53	-0.48	9.83
	Right parietal CPT	FA	1	45	2.13	0.1515	0.27	10.50	3.12	4.22	16.80	4.05	3.16	-2.32	10.40
		MD	1	45	0.13	0.7184	0.18	-2.81	2.08	-7.00	1.38	-3.87	2.09	-8.08	0.33
	Left occipital CPT	FA	1	39	4.67	0.0369	0.18	-2.28	3.27	-8.90	4.34	12.06	5.75	0.43	23.69
		MD	1	39	0.00	0.9585	0.10	0.58	1.48	-2.41	3.57	0.84	4.67	-8.62	10.29
	Right occipital CPT	FA	1	45	0.08	0.7832	0.19	19.40	8.82	1.65	37.20	16.40	9.73	-3.21	36.00
		MD	1	45	8.44	0.0057	0.34	-0.55	1.66	-3.89	2.78	-7.35	1.66	-10.70	-3.99
	Right nd-DRTC	FA	1	44	1.99	0.1656	0.13	2.08	2.26	-2.48	6.65	-2.21	2.32	-6.87	2.46
		MD	1	44	8.37	0.0059	0.22	-1.03	0.68	-2.39	0.34	2.03	0.83	0.36	3.70
	Left nd-DRTC	FA	1	45	0.00	0.9646	0.10	1.75	4.11	-6.53	10.00	2.01	4.12	-6.28	10.30
		MD	1	45	4.59	0.0377	0.21	0.68	2.58	-4.51	5.87	-7.17	2.63	-12.46	-1.88

ASHAFACS (CI)

tDCS Group (Anode)															
DV	Tract	DTI	nteraction (Treatment * Tract					tDCS				Sham			
			df1	df2	F	p	R^2	b	SE	CI.low	CI.high	b	SE	CI.low	CI.high
ASHAFACS (CI)	Left frontal CPT	FA	1	24	1.29	0.2668	0.34	###	1.44	-3.26	2.69	1.80	1.31	-0.91	4.51
		MD	1	24	1.98	0.1721	0.36	0.26	0.86	-1.52	2.03	-1.30	0.77	-2.89	0.28
	Right frontal CPT	FA	1	42	0.13	0.7181	0.35	0.61	1.30	-2.02	3.23	-0.01	1.14	-2.31	2.29
		MD	1	42	1.61	0.2112	0.37	0.43	0.93	-1.45	2.31	-1.06	0.88	-2.84	0.72
	Left parietal CPT	FA	1	36	0.72	0.4025	0.36	0.06	1.20	-2.38	2.49	-1.22	1.14	-3.53	1.09
		MD	1	36	0.08	0.7831	0.35	0.15	0.52	-0.90	1.19	0.31	0.50	-0.69	1.32
	Right parietal CPT	FA	1	42	1.81	0.1854	0.37	1.70	1.99	-2.31	5.72	-1.67	1.84	-5.38	2.05
		MD	1	42	0.96	0.3321	0.36	0.22	1.23	-2.26	2.70	-1.24	1.18	-3.61	1.14
	Left occipital CPT	FA	1	31	6.29	0.0176	0.48	0.48	0.85	-1.25	2.22	-2.27	0.82	-3.94	-0.61
		MD	1	31	6.06	0.0196	0.46	-0.11	0.32	-0.76	0.55	0.79	0.32	0.13	1.45
	Right occipital CPT	FA	1	42	6.06	0.0180	0.43	-0.85	1.57	-4.03	2.33	3.91	1.54	0.81	7.01
		MD	1	42	2.06	0.1588	0.38	0.01	0.64	-1.27	1.30	-1.07	0.62	-2.32	0.18
	Right nd-DRTC	FA	1	36	1.03	0.3160	0.38	0.03	1.19	-2.38	2.45	-1.51	1.14	-3.81	0.79
		MD	1	36	0.50	0.4831	0.35	0.39	0.57	-0.76	1.54	-0.02	0.56	-1.15	1.10
	Left nd-DRTC	FA	1	42	7.26	0.0101	0.43	2.32	1.73	-1.17	5.80	-3.83	1.62	-7.11	-0.55
		MD	1	42	0.48	0.4937	0.35	0.67	1.28	-1.92	3.25	-0.50	1.27	-3.06	2.07

tDCS Group (Cathode)																
DV	Tract	DTI	Interaction (Treatment * Tract)					tDCS				Sham				
			df1	df2	F	p	R^2	b	SE	CI.low	CI.high	b	SE	CI.low	CI.high
ASHAFACS (CI)	Left frontal CPT	FA	1	16	15.14	0.0013	0.61	7.63	1.95	3.49	11.78	2.96	1.95	-1.18	7.11
		MD	1	16	3.56	0.0775	0.36	-1.90	0.88	-3.76	-0.03	0.38	0.88	-1.48	2.24
	Right frontal CPT	FA	1	44	2.06	0.1584	0.06	1.73	1.54	-1.37	4.84	-1.34	1.55	-4.47	1.78
		MD	1	44	1.92	0.1729	0.08	0.07	0.96	-1.87	2.01	-1.81	0.97	-3.77	0.14
	Left parietal CPT	FA	1	31	6.09	0.0193	0.27	0.98	0.98	-1.02	2.97	-2.26	0.97	-4.24	-0.29
		MD	1	31	0.40	0.5302	0.15	-0.80	1.21	-3.28	1.67	0.33	1.30	-2.31	2.97
	Right parietal CPT	FA	1	44	2.91	0.0953	0.07	1.96	1.59	-1.24	5.15	-1.88	1.61	-5.12	1.37
		MD	1	44	0.38	0.5422	0.03	0.50	1.02	-1.55	2.56	-0.38	1.02	-2.44	1.68
	Left occipital CPT	FA	1	38	7.83	0.0080	0.21	-0.03	1.47	-3.00	2.93	-8.34	2.58	-13.60	-3.13
		MD	1	38	7.04	0.0116	0.18	-0.28	0.64	-1.58	1.02	-5.95	2.03	-10.06	-1.84
	Right occipital CPT	FA	1	44	8.11	0.0067	0.20	0.19	4.10	-8.09	8.46	#####	4.35	-22.91	-5.39
		MD	1	44	0.01	0.9421	0.06	-0.94	0.89	-2.73	0.85	-1.03	0.89	-2.83	0.77
	Right nd-DRTC	FA	1	43	6.97	0.0115	0.13	-1.83	1.02	-3.90	0.23	1.77	1.04	-0.33	3.87
		MD	1	43	13.40	0.0007	0.22	0.70	0.31	0.07	1.32	-1.06	0.37	-1.81	-0.30
	Left nd-DRTC	FA	1	44	2.21	0.1447	0.07	-1.01	1.92	-4.88	2.87	3.00	1.89	-0.81	6.81
		MD	1	44	1.64	0.2069	0.09	-0.17	1.25	-2.69	2.34	-2.45	1.27	-5.01	0.12

ASHAFACS (QDC)

tDCS Group (Anode)																
DV	Tract	DTI	Interaction (Treatment * Tract)					tDCS				Sham				
			df1	df2	F	p	R^2	b	SE	CI.low	CI.high	b	SE	CI.low	CI.high
ASHAFACS (QDC)	Left frontal CPT	FA	1	24	21.45	0.0001	0.64	6.15	1.27	3.53	8.78	###	1.16	-3.76	1.03
		MD	1	24	26.02	<.0001	0.63	-2.72	0.77	-4.32	-1.12	2.35	0.69	0.93	3.77
	Right frontal CPT	FA	1	42	5.36	0.0256	0.37	3.62	1.68	0.23	7.01	-1.47	1.47	-4.45	1.50
		MD	1	42	0.70	0.4069	0.38	1.58	1.21	-0.86	4.02	2.86	1.14	0.55	5.17
	Left parietal CPT	FA	1	36	0.48	0.4924	0.38	0.63	1.54	-2.50	3.75	-0.71	1.46	-3.68	2.26
		MD	1	36	1.66	0.2053	0.52	1.12	0.58	-0.05	2.30	2.00	0.56	0.87	3.13
	Right parietal CPT	FA	1	42	2.67	0.1097	0.34	5.01	2.66	-0.35	10.37	-0.45	2.46	-5.42	4.51
		MD	1	42	1.16	0.2879	0.31	-0.89	1.67	-4.27	2.48	1.28	1.60	-1.95	4.51
	Left occipital CPT	FA	1	31	3.11	0.0879	0.54	-3.21	1.00	-5.25	-1.17	-0.94	0.96	-2.89	1.02
		MD	1	31	0.80	0.3770	0.70	1.70	0.30	1.09	2.31	1.39	0.30	0.78	2.01
	Right occipital CPT	FA	1	42	1.46	0.2337	0.32	2.20	2.26	-2.36	6.77	-1.15	2.21	-5.61	3.30
		MD	1	42	3.70	0.0614	0.34	-1.08	0.86	-2.81	0.66	0.89	0.84	-0.80	2.58
	Right nd-DRTC	FA	1	36	0.06	0.8069	0.37	-0.14	1.54	-3.27	2.99	-0.63	1.47	-3.61	2.36
		MD	1	36	2.84	0.1006	0.48	0.87	0.66	-0.47	2.21	2.01	0.65	0.70	3.33
	Left nd-DRTC	FA	1	42	2.43	0.1266	0.38	-6.13	2.37	-10.91	-1.36	-1.26	2.23	-5.75	3.24
		MD	1	42	0.02	0.9038	0.39	3.51	1.63	0.21	6.81	3.25	1.62	-0.02	6.52

tDCS Group (Cathode)																
**DV**	**Tract**	**DTI**	**Interaction (Treatment * Tract**					**tDCS**				**Sham**				
			**df1**	**df2**	**F**	**p**	**R^2**	**b**	**SE**	**CI.low**	**CI.high**	**b**	**SE**	**CI.low**	**CI.high**
ASHAFACS (QDC)	Left frontal CPT	FA	1	16	14.12	0.0017	0.74	9.62	1.46	6.54	12.71	6.26	1.46	3.17	9.35
		MD	1	16	20.63	0.0003	0.70	-3.37	0.55	-4.53	-2.21	0.06	0.55	-1.11	1.22
	Right frontal CPT	FA	1	44	4.72	0.0352	0.38	3.15	1.08	0.97	5.34	-0.13	1.09	-2.33	2.08
		MD	1	44	1.36	0.2505	0.34	-1.53	0.71	-2.95	-0.10	-0.36	0.71	-1.80	1.07
	Left parietal CPT	FA	1	31	0.64	0.4311	0.32	0.74	0.87	-1.04	2.51	-0.19	0.86	-1.95	1.56
		MD	1	31	4.37	0.0449	0.52	-3.41	0.84	-5.12	-1.69	-0.83	0.90	-2.65	1.00
	Right parietal CPT	FA	1	44	1.48	0.2307	0.31	1.86	1.19	-0.53	4.25	-0.18	1.20	-2.61	2.24
		MD	1	44	0.05	0.8257	0.28	-0.25	0.76	-1.77	1.28	-0.49	0.76	-2.02	1.05
	Left occipital CPT	FA	1	38	0.01	0.9153	0.29	-0.80	1.16	-3.16	1.55	-1.06	2.04	-5.19	3.08
		MD	1	38	2.21	0.1451	0.46	-1.29	0.44	-2.18	-0.39	-3.46	1.39	-6.28	-0.65
	Right occipital CPT	FA	1	44	2.26	0.1401	0.35	-2.21	3.21	-8.67	4.26	-8.12	3.40	-14.97	-1.27
		MD	1	44	0.92	0.3422	0.30	-0.79	0.67	-2.14	0.56	0.12	0.67	-1.24	1.47
	Right nd-DRTC	FA	1	43	0.04	0.8466	0.37	1.28	0.76	-0.25	2.81	1.08	0.77	-0.47	2.63
		MD	1	43	0.16	0.6880	0.44	-0.66	0.23	-1.12	-0.21	-0.52	0.27	-1.07	0.03
	Left nd-DRTC	FA	1	44	1.39	0.2442	0.31	-0.21	1.44	-3.11	2.69	2.17	1.41	-0.68	5.01
		MD	1	44	0.36	0.5507	0.29	-0.01	0.95	-1.93	1.91	-0.83	0.97	-2.79	1.13
